# Combined Multistate and Kohn-Sham Density Functional Theory Studies of the Elusive Mechanism of *N*-Dealkylation of *N,N*-Dimethylanilines Mediated by the Biomimetic Nonheme Oxidant Fe^IV^(O)(N4Py)(ClO_4_)_2_

**DOI:** 10.3389/fchem.2018.00406

**Published:** 2018-09-10

**Authors:** Lili Yang, Xin Chen, Zexing Qu, Jiali Gao

**Affiliations:** ^1^Laboratory of Theoretical and Computational Chemistry, Institute of Theoretical Chemistry, Jilin University, Changchun, China; ^2^Department of Chemistry, University of Minnesota, Minneapolis, MN, United States

**Keywords:** C-H activation, N-dealkylation, mechanism, MSDFT, HAT, CEPT

## Abstract

The oxidative C-H bond activation mediated by heme and nonheme enzymes and related biomimetics is one of the most interesting processes in bioinorganic and oxidative chemistry. However, the mechanisms of these reactions are still elusive and controversy due to the involvement of highly reactive metal-oxo intermediates with multiple spin states, despite extensive experimental efforts, especially for the N-dealkylation of N,N-dialkyalinines. In this work, we employed multistate density functional theory (MSDFT) and the Kohn-Sham DFT to investigate the mechanism of N-demethylation of N,N-dimethyalinines oxidized by the reaction intermediate Fe^IV^(O)(N4Py)(ClO_4_)_2_. The Kohn-Sham DFT study demonstrated that the reaction proceeds via a rate-limiting hydrogen atom transfer (HAT) step and a subsequent barrier-free oxygen rebound step to form the carbinol product. The MSDFT investigation on the first C-H activation further showed that this step is an initial hydrogen atom abstraction that is highly correlated between CEPT and HAT, i.e., both CEPT and HAT processes make significant contributions to the mechanism before reaching the diabatic crossing point, then the valence bond character of the adiabatic ground state is switched to the CEPT product configuration. The findings from this work may be applicable to other hydrogen abstraction process.

## Introduction

**Scheme 1:**
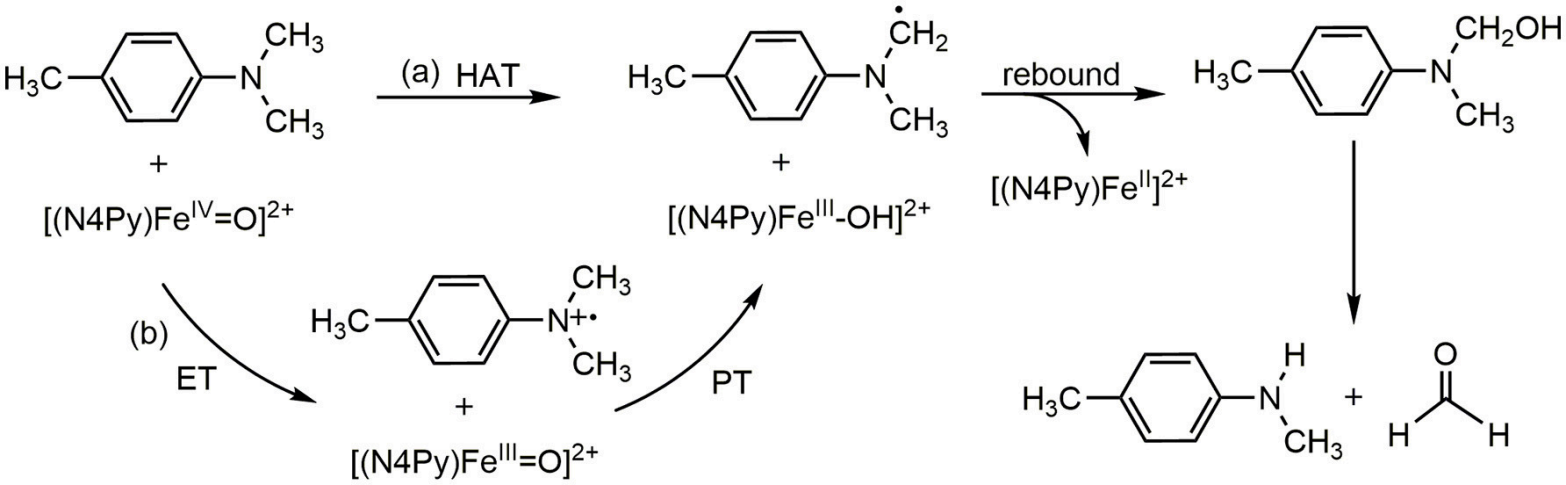
ET-PT(SET) and HAT mechanism in the para-substituted N,N-dimethylanilines activated by [(N4Py)-Fe^IV^ = O](ClO_4_)_2_.

Heme and nonheme iron enzymes mediate a variety of fundamental biochemical transformations which are vital to biological processes. These enzymes are found in all aerobic species (Ortiz de Montellano and De Voss, 2002) and carry out a myriad of significant catalytic transformations, ranging from detoxification, biosynthesis to drug metabolism (Nam, 2007; Li D. et al., 2012). In particular, hydrogen atom transfer (HAT) mediated by high-valent iron(IV)-oxo complexes is a key process in the activation of C-H, O-H, or N-H bonds (Wang et al., 2007). Over the years,

the mechanism of hydrogen abstraction has been actively investigated because of its vital function (Mayer et al., 2002; Jeong et al., 2008; Tishchenko et al., 2008; Mayer, 2011; Sirjoosingh and Hammes-Schiffer, 2011; Cembran et al., 2012; Lai et al., 2012; Usharani et al., 2013; Saouma and Mayer, 2014), including N-dealkylation reactions. As shown in Scheme 1, the reaction proceeds via an initial hydrogen abstraction, followed by an oxygen rebound step to form a carbinolamine intermediate. Then, after nonenzymatic and water-assisted C-N bond cleavage, the final products of an aldehyde and a secondary amine are produced. In fact, the mechanism of the initial hydrogen transfer step has been studied quite thoroughly (Wimalasena and May, 1987; Bhakta and Wimalasena, 2005; Nehru et al., 2007; Chiavarino et al., 2008; Li C. et al., 2009; Li D. et al., 2009 Baciocchi et al., 2010; Roberts and Jones, 2010; Wang et al., 2010; Park et al., 2011, 2014; Morimoto et al., 2012; Barbieri et al., 2015). Evidence exists to suggest that the first step undergoes a single electron transfer (SET) process, namely an electron transfer from the aniline to the enzyme and produce an amino radical intermediate after the deprotonation of the aniline cation, as Pathway (a) shown in the Scheme 1 (Wimalasena and May, 1987; Bhakta and Wimalasena, 2005; Baciocchi et al., 2010; Park et al., 2011; Barbieri et al., 2015). However, some researchers suggested that this step is a HAT process, Pathway (b) shown in Scheme 1 (Li C. et al., 2009; Li D. et al., 2009; Roberts and Jones, 2010). At the center of this seemingly controversy underscores the distinction between concerted and stepwise mechanisms for electron transfer and the proton transfer. The overall reaction is a proton-coupled electron transfer (PCET), which can be a HAT or a concerted-asynchronous proton-electron transfer (CEPT) (Hammes-Schiffer, 2001, 2015; Mayer et al., 2002; Hammes-Schiffer and Iordanova, 2004; Reece et al., 2006; Rhile et al., 2006; Huynh and Meyer, 2007; Hammes-Schiffer and Soudackov, 2008; Jeong et al., 2008; Tishchenko et al., 2008; Reece and Nocera, 2009; Hammes-Schiffer and Stuchebrukhov, 2010; Sirjoosingh and Hammes-Schiffer, 2011; Cembran et al., 2012; Lai et al., 2012; Usharani et al., 2013; Park et al., 2014; Saouma and Mayer, 2014). Nehru et al. (2007) firstly elucidated the N-dealkylation of N,N-dimethylaniline mediated by heme and synthetic nonheme oxo-iron(IV) complexes. In their experiments, various substituted N,N-dimethylanilines are used as probes and clarified the C-H abstraction in N-dealkylation is a rate-limiting electron transfer (ET) followed by a proton transfer (PT) process. However, the inter and intramolecular kinetic isotope effect (KIE) experiments demonstrated that the ET process may occur by coupling with the PT process but these two processes are not kinetically independent (Nehru et al., 2007). That is to say, the N-dealkylation process may go through a concerted PCET process. Thus, we decide to carry out theoretical calculation of this system to solve this controversy.

For reactions involving coupled transfers of an electron and a proton, it is useful to characterize the reaction coordinate using diabatic potential energy surfaces that define the localization of the proton and electron in their donor and acceptor sites. Kohn-Sham density functional theory (DFT) based on the Born Oppenheimer approximation with delocalized orbitals is not appropriate to model these diabatic states. On the other hand, a method based on valence bond theory (Hiberty et al., 1992; Cooper, 2002; Song et al., 2005) can effectively describe charge-localized configurations (Shaik et al., 2008; Lai et al., 2012; Usharani et al., 2013, 2014). Shaik et al. used a valence bond (VB) model to represent the mechanisms of C–H bond activation and O-H/N-H bond activation (Usharani et al., 2013). The XMVB program can be used to define the diabatic states (Song et al., 2005). Using a local determinant representation of individual Lewis structures, which effectively contracts many VB configurations into a single determinant approximation, we introduced a mixed molecular orbital and valence bond (MOVB) model, in which the block-localized wave (BLW) function method is used to define diabatic electronic states (Song et al., 2009; Gao et al., 2010; Mo et al., 2011; Cembran et al., 2012). This idea has been extended to density functional theory, and the general approach is called multistate density functional theory (MSDFT), and it can be used to study the mechanisms of PCET processes (Song et al., 2009; Gao et al., 2010; Mo et al., 2011; Cembran et al., 2012). To this end, dynamic correlation is first incorporated into the definition of the diabatic states in the active space relevant to PCET reactions. Then, static correlation is taken into account by configuration interaction of these diabatic states to yield the adiabatic electronic states. Consequently, MSDFT follows a dynamic-then-static ansatz, taking advantage of both wave function theory and density functional theory (Song et al., 2009; Gao et al., 2010; Mo et al., 2011; Cembran et al., 2012). Thus, the MSDFT has been successfully applied in the construction of adiabatic and diabatic energy surfaces of coupled proton and electron transfer process in the isoelectronic series, HNO_3_ in aqueous solution and the hydrogen molecule dissociation (Cembran et al., 2012; Gao et al., 2016; Ren et al., 2016; Grofe et al., 2017a,b).

In this article, we use Kohn-Sham DFT and MSDFT to study the pathway of hydrogen abstraction from para-substituted N,N-dimethylanilines by [(N4Py)-Fe^IV^ = O](ClO_4_)_2_, and the subsequent steps. Our traditional Kohn-Sham DFT calculations show that the mechanism of the C-H abstraction is a HAT mechanism, whereas MSDFT reveals that the mechanism is a blended of HAT and CEPT mechanism to be exactly from the effective diabatic energy surfaces of HAT and CEPT, as pathway (c) shown in Scheme 1. Since the individual reaction steps can be separately defined using MSDFT, it is possible to provide a definitive answer to the mechanistic debate about the hydrogen abstraction reaction between N,N-dimethylaniline and a heme or a synthetic nonheme oxo-iron(IV) complexes.

## Theoretical methods

In this study, the [(N4Py)-Fe^IV^ = O](ClO_4_)_2_ and para-substituted N,N-dimethylanilines were used as the active species of a biomimetic nonheme oxidant and the substrate (Scheme 1), respectively. Both the low-spin (LS) triplet and high-spin (HS) quintet states of nonheme oxidant were considered.

Kohn-Sham DFT calculations were performed using the Gaussian 09 program (Frisch et al., 2010). Following early studies, the B3LYP functional, which has been successfully applied to the Fe compounds (Kumar et al., 2005; Shaik et al., 2005, 2007, 2008; Hirao et al., 2006; Wang et al., 2007; Tishchenko et al., 2008; Usharani et al., 2013), was chosen for all the calculations. Considering the computational cost, the basis set with LANL2DZ for Fe and 6-31G(d,p) for all remaining atoms (B1) was used in geometry optimizations, frequency and the intrinsic reaction coordinate (IRC) calculations. The basis for nonmetal atoms were expanded to 6-311++G(d,p) (B2) in single-point energy calculations, which along with zero-point energy corrections were used in all discussions in the text. Mulliken spin densities and NBO charges were analyzed to gain insights into the electronic properties of the key reaction species

**Scheme 2:**
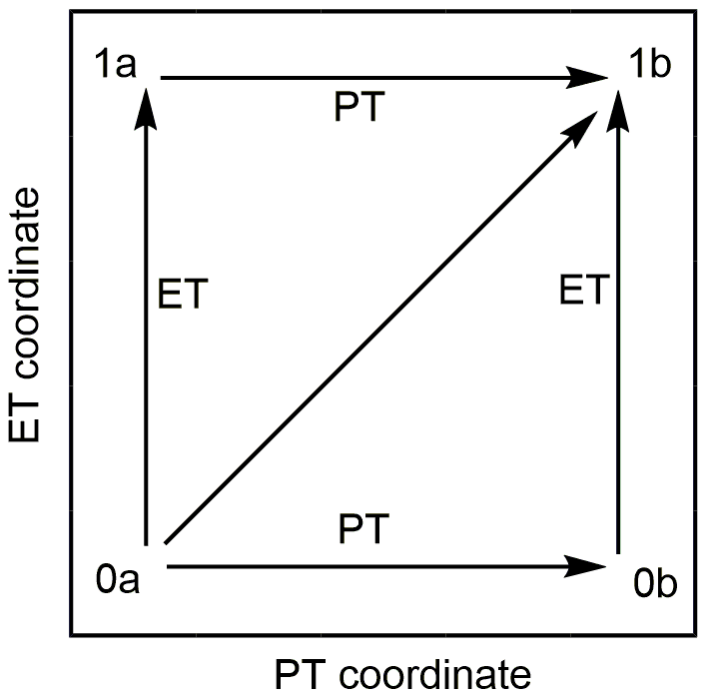
More O'Ferrall–Jencks diagram for the H-abstraction reaction of para-substituted N,N-dimethylanilines mediated by [(N4Py)-Fe^IV^ = O](ClO_4_)_2_. The horizontal and vertical coordinates stand for proton transfer (PT) and electron transfer (ET) pathway, and the diagonal line implies the concerted PT-CT pathway. The four corners (0a, 1a, 0b, 1b) of the diagram stand for the reactant state, electron transfer state, proton transfer state, and product state, respectively.

The MSDFT calculations were carried out using a locally modified GAMESS package (Schmidt et al., 1993; Song et al., 2005, 2009). Scheme 2 illustrates a More O'Ferrall–Jencks diagram (O'Ferrall, 1970) for the electron transfer and proton transfer pathways of the C-H activation of N-dealkylation by [(N4Py)-Fe^IV^ = O](ClO_4_)_2_, in which the four corners depict the diabatic electronic states, corresponding to the reactant (lower left) and product (upper right) states, and the electron transfer (upper left) and proton transfer (lower right) intermediate. In the MSDFT framework, they were defined and optimized using block localized Kohn-Sham (BLKS) DFT (Cembran et al., 2012)

Reactant compound (0a),

(1) $$\Psi_{0a}^{BLKS}=\hat{A}\{\Omega_{0a}^{1}(Sub)\Omega_{0a}^{2}(LFe^{IV}=OX_{2})\}$$

Proton transfer state (0b),

(2) $$\Psi_{0b}^{BLKS}=\hat{A}\{\Omega_{0b}^{1}\left(Sub^{-}\right)\Omega_{0b}^{2}\left(LFe^{IV}-OHX_{2}^{+}\right)\}$$

Charge transfer state (1a),

(3) $$\Psi_{1a}^{BLKS}=\hat{A}\{\Omega_{1a}^{1}\left(Sub^{+}\right)\Omega_{1a}^{2}\left(LFe^{III}=OX_{2}^{-}\right)\}$$

Product compound (1b),

(4) $$\Psi_{1b}^{BLKS}=\hat{A}\{\Omega_{1b}^{1}\left(Sub^{\cdot }\right)\Omega_{1b}^{2}\left(LFe^{III}=OHX_{2}\right)\}$$

where Ã, is the antsymmetrizer, $\Omega_{\gamma }^{\text{k}}$ is the product of the occupied BLKS orbitals of the *k*th(*k* = 1, 2) fragment defined in the diabatic stateγ (γ = 0a, 0b, 1a, and 1b), L, X and Sub stand for N4Py, ${\text{ClO}}_{4}^{-}$ and the para-substituted N,N-dimethylanilines. Equations (1)–(4) constitute a contracted active space in MSDFT, which can be used in configuration interaction to yield the adiabatic ground and excited states potential energy surfaces:

(5) $$\Phi_{MSDFT}^{GS}=\sum_{ip}c_{ip}^{GS}\Psi_{ip}^{BLKS}(c_{ip};i=0,1;p=a,b)$$

(6) $$\Phi_{MSDFT}^{ES}=\sum_{ip}c_{ip}^{ES}\Psi_{ip}^{BLKS}\;(c_{ip};i=0,1;p=a,b)$$

Thus, the potential energy surfaces for the overall coupled PT and ET processes, either concerted or stepwise, are represented as the admixture of the four basis configurations.

To elucidate the mechanistic origin of N-H bond activation beyond the orbital picture, we employed MSDFT to characterize the HAT and CEPT reaction pathways. In MSDFT framework, the two-state representation of the HAT and CEPT mechanism can be constructed from the four diabatic states in Scheme 2 according to Equations (7)–(10) (Cembran et al., 2012).

(7) $$\Phi_{R}^{CPET}=c_{0a}\Psi_{0a}^{BLKS}+c_{0b}\Psi_{0b}^{BLKS}$$

(8) $$\Phi_{P}^{CPET}=c_{1a}\Psi_{1a}^{BLKS}+c_{1b}\Psi_{1b}^{BLKS}$$

(9) $$\Phi_{R}^{HAT}=c_{0a}\Psi_{0a}^{BLKS}+c_{1a}\Psi_{1a}^{BLKS}$$

(10) $$\Phi_{P}^{HAT}=c_{0b}\Psi_{0b}^{BLKS}+c_{1b}\Psi_{1b}^{BLKS}$$

where the coefficients are determined by separate (2 × 2) configuration interactions involving the two diabatic states in each equation.

Equations (7) and (8) describe the reactant and product states in the CEPT mechanism, respectively. Here, the Born-Oppenheimer approximation breaks down and multi-configuration methods are needed. For HAT mechanism, which distinguishes from CEPT by strong electronic coupling to result in a strongly avoided crossing with the ground and excited states well-separated, the Born-Oppenheimer approximation is fully valid and the electronic structure is stationary with respect to the proton nuclear coordinates. Thus, the wave functions for the reactant and product states of the HAT mechanism can be expressed as linear combinations of the electronic configurations with the transferring proton localized on the donor and acceptor sites, respectively (Equations 9, 10).

## Result and discussion

**Figure 1:**
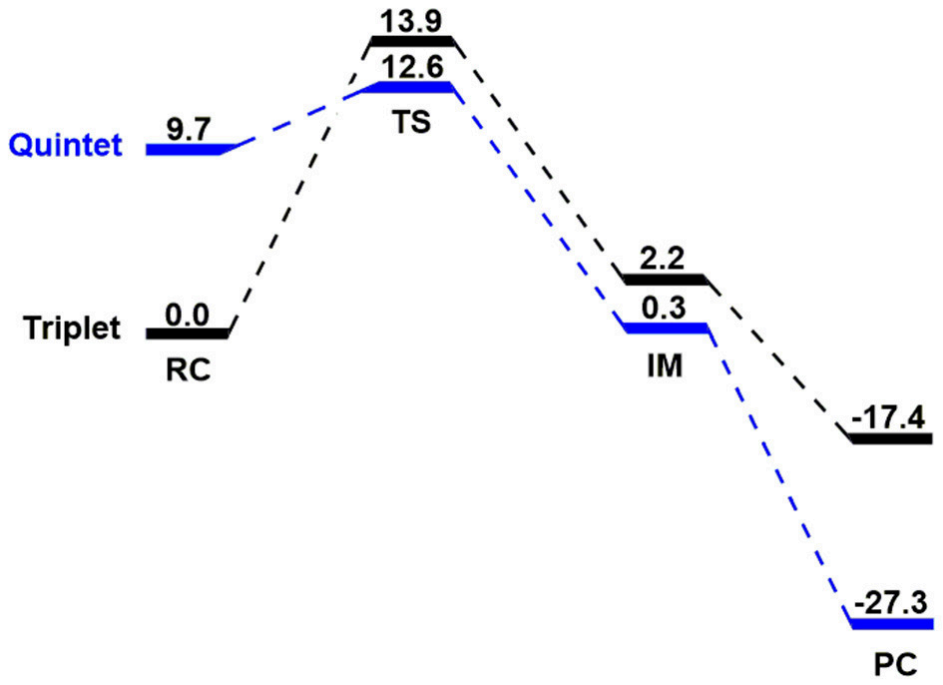
The triplet and quintet energy profiles (in kcal/mol) related to triplet reactant state N-dealkylation of para-substituted N,N-dimethylanilines activated by [(N4Py)-Fe^IV^ = O](ClO_4_)_2_.

Kohn-Sham DFT calculations on the hydroxylation of N,N-dimethylaniline oxidized by the reactive species Fe^IV^(O)(N4Py)(ClO_4_)_2_ on the triplet and quintet spin states are first presented in Figure 1. Then, MSDFT results on the oxidative C-H bond activation is introduced to elaborate the mechanistic origin of the formally PCET process. We found in Figure 1 that the ground state of the reagent complex (RC) is the triplet spin state, whereas the quintet state lies 9.7 kcal mol^−1^ higher in energy. Interestingly, the transition state (TS) for the hydrogen abstraction on the adiabatic potential energy surface is switched to a quintet spin-state, which is about 1 kcal/mol lower than that in the triplet state. The overall barriers, relative to the triplet RC configuration are 12.6 and 13.9 kcal/mol for the two spin states, respectively. Obviously, this is a two-state reactivity (TSR) that was originally proposed by Shaik et al. (Schröder et al., 2000; Shaik et al., 2005, 2007; Hirao et al., 2006; Klinker et al., 2009). The nascent intermediate (IM) lies 0.3 kcal mol-1 on the quintet state and for the triplet IM, 2.2 kcal mol^−1^. The subsequent oxygen recombination step is a barrierless, exothermic process, and the product complexes (PCs) lies −27.3 kcal mol^−1^ for the quintet PC and −17.4 kcal mol^−1^ for the triplet one.

**Figure 2:**
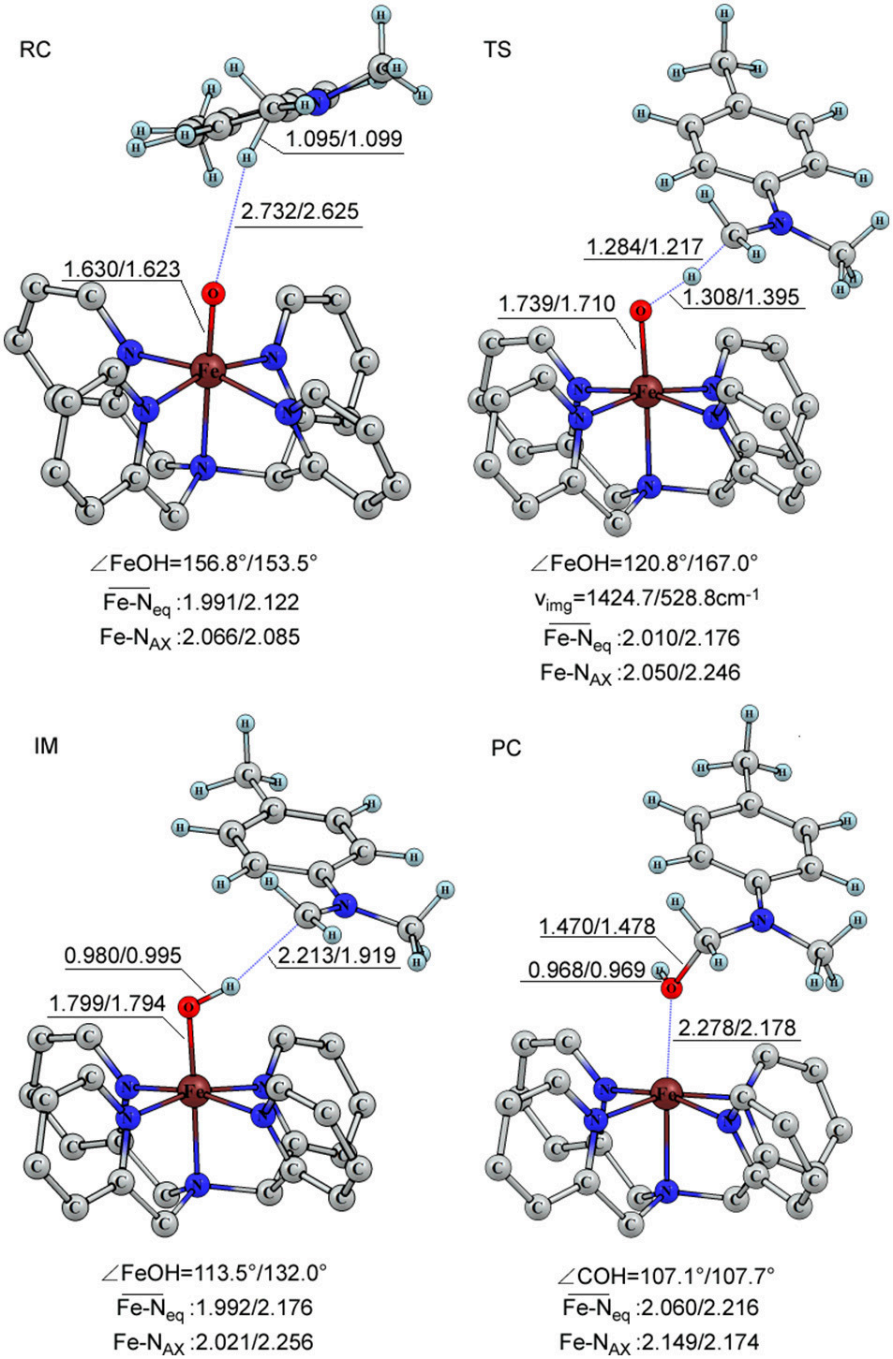
The important bond length (in Å), angle (in degree) and vibration frequency (in cm^−1^) of the key intermediates for the triplet and quintet in the C-H bond activation of para-substituted N,N-dimethylanilines catalyzed by [(N4Py)-Fe^IV^ = O](ClO_4_)_2_.

The geometric information of these reaction intermediates is presented in Figure 2. For the triplet reagent complex, the C-H bond of the substrate is 1.095 Å, Fe = O is 1.630 Å and the distance between H(C) and O(Fe) is 2.732 Å. The bond angle (∠ H-O-Fe) is 156.8°. For the quintet transition state, the C-H length becomes 1.284 Å, Fe = O 1.739 Å and H(C) and O(Fe) 1.308 Å, and the bond angle (∠ H-O-Fe) becomes 120.8°. At the intermediate state, the Fe-OH bond is elongated to 1.799 Å, a formally a single bond, and the bond angle (∠ H-O-Fe) is changed to 113.5°. KS-DFT results correspond to a synchronous transfer of the proton and electron in the first reaction step. Following the hydrogen abstraction, an oxygen rebound step occurs, the O-H group is transferred to the C radical site. At the product complex state, the length of C-O bond is 1.470 Å.

For the quintet state, there is little difference in structure from that of the triplet for the reactant species, with key structural parameters shown in Figure 2. For the quintet transition state, the key structural parameters show some variation with slightly shorter C-H (1.217 Å) and the Fe = O (1.710 Å) bond lengths, and slightly longer H(C)-O(Fe) distance (1.395 Å) compared to the corresponding data of the triplet. The most striking structural variation is the ∠ H-O-Fe bond angle at 167.0°, which is 53.5° greater than the triplet counterpart. In addition, the imaginary vibration frequency at the TS for the quintet is reduced by a factor of two relative to that of the triplet transition state. Whereas, for the quintet transition state, the contribution of the Fe = O stretching vibration and the substrate swinging vibrations to the imaginary vibration more than that those on the triplet transition state. Furthermore, the longer Fe = O bond length at the transition state, accompanied by a shorter H-O(Fe) distance, indicate that the TS is more advanced toward the product side than that in the triplet state, and that a greater degree of electron transfer to the iron center. As for C-O bond, the length of the triplet is slightly shorter than that of quintet, consistent with Shaik et al. (Klinker et al., 2009). For the C-H and O-H distance. compared to that of the quintet one, the length of the C-H distance is longer and the O-H one is shorter for the triplet transition state (Hirao et al., 2006).

The segmental spin densities and Mulliken charges of the reactants, transition states and products of the triplet and quintet are shown in Table 1. Herein, the Sub-H stands for the para-substituted N,N-dimethylanilines without the proton of the C-H bond oxidated in the reaction, of which the spin densities are all 0.00 for reactants. when getting to the triplet transition state, the value becomes 0.57. Meanwhile, the spin density of Fe = O unit is reduced by 0.56, and there is no net change in spin density on the ligand N4Py and perchlorate ions. Thus, the β electron transfers to the singly occupied orbital of Fe = O unit, pairing with the existing α electron. At the product complex, the spin density of Sub-H is 0.96, indicating that almost the entire β electron has been transferred. Concomitantly, the spin density of the Fe = O unit is decreased by 0.94, revealing that the β electron spin density is transferred to the Fe = O unit. For the quintet transition state, the spin density of Sub-H changes from 0.00 to −0.58, reduced by 0.56, and that of Fe = O is increased by 0.47, suggesting that the α electron spin density is shifted from Sub, mostly to empty orbital of Fe = O unit. For the product complex, the spin density of the Sub-H is further reduced to −0.80, as that of the Fe = O unit is increased to 4.31. Thus, from the transition state to the product, the entire α electron spin density is transferred to the Fe = O unit.

**Table 1:** Mulliken charges and spin densities of main intermediates in the H-transfer reaction of para-substituted N,N-dimethylanilines mediated by [(N4Py)-Fe^IV^ = O](ClO_4_)_2_, herein, there is no changes in the spin densities and charges of the counterions ${\text{ClO}}_{4}^{-}$, Sub denotes the para-substituted N,N-dimethylanilines and Sub-H denotes the para-substituted N,N-dimethylanilines without H atom.

			**Spin density (*****ρ*****)**					**Charge (*****Q*****)**	
	**Fe**	**O**	**N_4_Py**	**H**	**Sub-H**	**Fe**	**O**	**N_4_Py**	**H**	**Sub-H**
^3^**RC**	1.16	0.88	−0.04	−0.00	0.00	0.90	−0.52	1.31	0.15	−0.12
^5^**RC**	2.97	0.70	0.33	−0.00	0.00	1.01	−0.52	1.20	0.15	−0.12
^3^**TS**	0.88	0.60	−0.04	−0.03	0.60	0.85	−0.67	1.11	0.31	0.07
^5^**TS**	3.80	0.34	0.44	−0.01	−0.57	1.03	−0.73	0.97	0.32	0.16
^3^**IM**	0.92	0.18	−0.10	0.02	0.94	0.81	−0.69	1.19	0.31	0.07
^5^**IM**	3.93	0.38	0.50	−0.02	−0.78	1.05	−0.78	0.96	0.36	0.13
^3^**PC**	1.96	0.01	0.02	0.00	0.01	0.67	−0.53	0.91	0.33	0.35
^5^**PC**	3.71	0.02	0.26	0.00	0.00	0.74	−0.55	0.83	0.34	0.37

**Figure 3:**
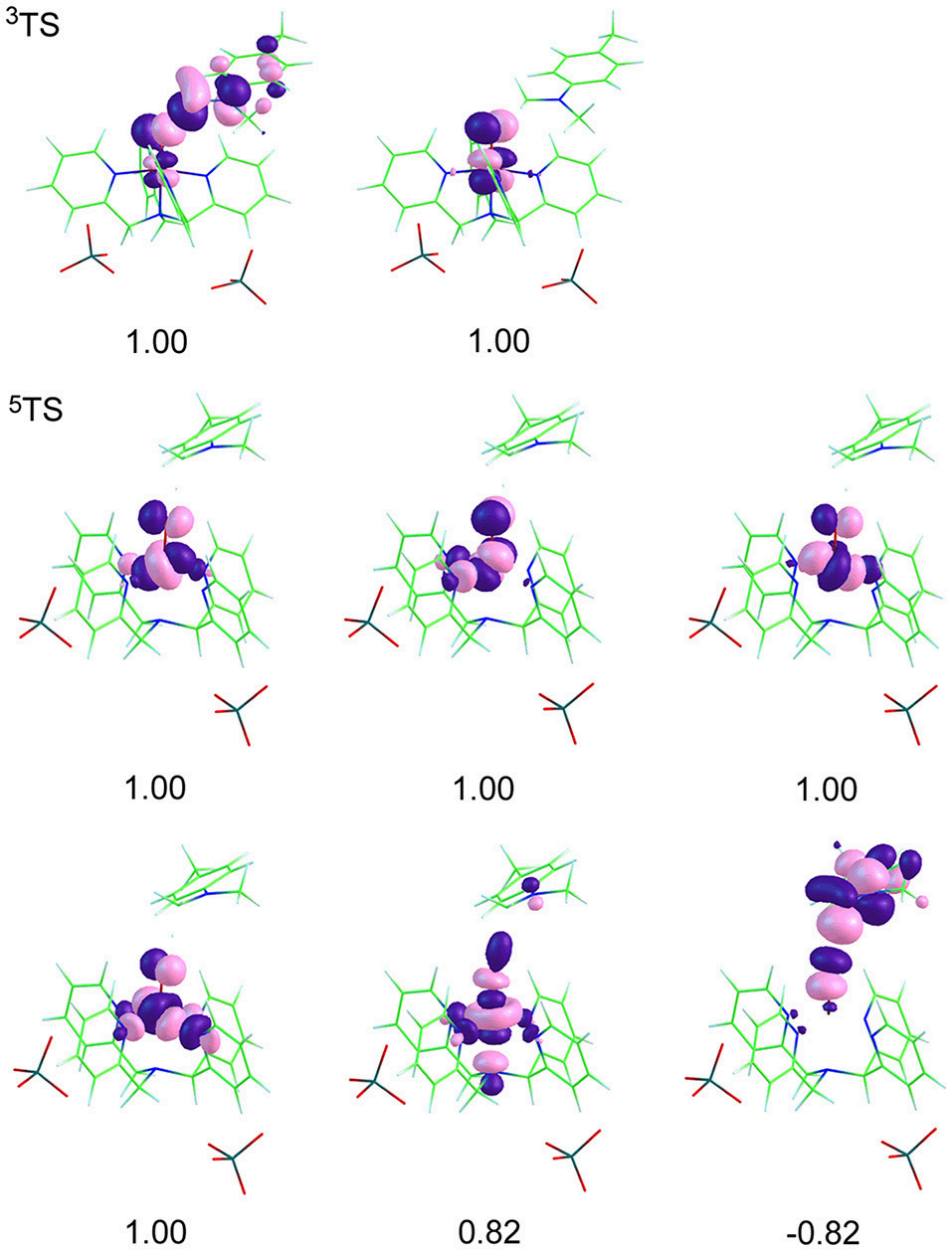
Spin natural orbitals (SNO) and their occupation numbers of main intermediates in the H-transfer reaction of para-substituted N,N-dimethylanilines mediated by [(N4Py)-Fe^IV^ = O](ClO_4_)_2_. A negative occupation number corresponds to spin β.

The spin natural orbitals (SNO) are shown in the Figure 3. According to the molecular orbital shape and electron configuration, these five d orbitals are spread in a distorted octahedron field (Kumar et al., 2005; Shaik et al., 2005, 2007, 2008; Hirao et al., 2006; Wang et al., 2007; Tishchenko et al., 2008; Usharani et al., 2013). The $\pi_{\text{xz}}^{*}$, $\pi_{\text{yz},}^{*}$ and $\sigma_{\text{z}2}^{*}$ antibonding orbitals are hybrid mainly by Fe(d) and O(p), in which, $\pi_{\text{xz}}^{*}$ and $\pi_{\text{yz}}^{*}$ are nearly in energy below $\sigma_{\text{z}2}^{*}$. Besides, Fe(d) and N(p) contribute to δ and $\sigma_{\text{xy}}^{*}$. Herein, the maximum direction of electron cloud belonging to Fe(d_xy_) is over against that of N(p), resulting in $\sigma_{\text{xy}}^{*}$ being of a high energy below $\sigma_{\text{z}2}^{*}$ and bonding orbital δ lies in the lowes. The way that an electron of triplet in δ orbital jumps to $\sigma_{\text{xy}}^{*}$ forms quintet makes the energy of quintet higher than that of triplet at initial reactant. But in turn, when a spin-up electron of σ_C−H_ transfers to the $\sigma_{\text{z}2}^{*}$ orbital, the five newly formed d orbitals in quintet possess d-d exchange interaction (Cartert and Goddard III, 1988a,b), this stabilizes the quintet, making it in a low energy. This is consistent with the energy profiles in Figure 1, a crossover occurs during the C-H activation, that's to say the balance in energy gets ready for the two-state reaction. At the reactant states of [(N4Py)-Fe^IV^ = O](ClO_4_)_2_, Fe contains four d electrons, its triplet has δ^2^π*^1^π*^1^ configuration, and the configuration of quintet is $\delta^{1}\pi {*}^{1}\pi {*}^{1}{\sigma {*}_{\text{xy}}}^{1}$. And at the transition states, a spin-up electron of σ_C−H_ transfers to the empty $\sigma_{\text{z}2}^{*}$ orbital on the quintet surface to activate the H-abstraction reaction, as can be seen from the molecular orbital shape, the included angle of the two orbitals is close to 180°, consistent with the value in Figure 2, namely, this α electron attacks the $\sigma_{\text{z}2}^{*}$ orbital almost head-on, in this way, a strong σ^*^ formed. Whereas, on the triplet surface, a β electron of σ_C−H_ transfers to the singly occupied π^*^ orbital during the reaction, the two orbitals forming an angle of about 120°, fitted with the result in Figure 2, that is, the β electron attacking the π^*^ orbital sideways. Compared with the two pathways, attacking head-on is easier and the formed σ^*^ is stronger, these contribute to the lower energy gap on the quintet surface than triplet. Furthermore, the electrons and protons of both triplet and quintet transfer via the direction of the σ-type nonbonding orbital which is along the C···H···O axis. This is a signature for HAT processes (Li C. et al., 2012).

**Figure 4:**
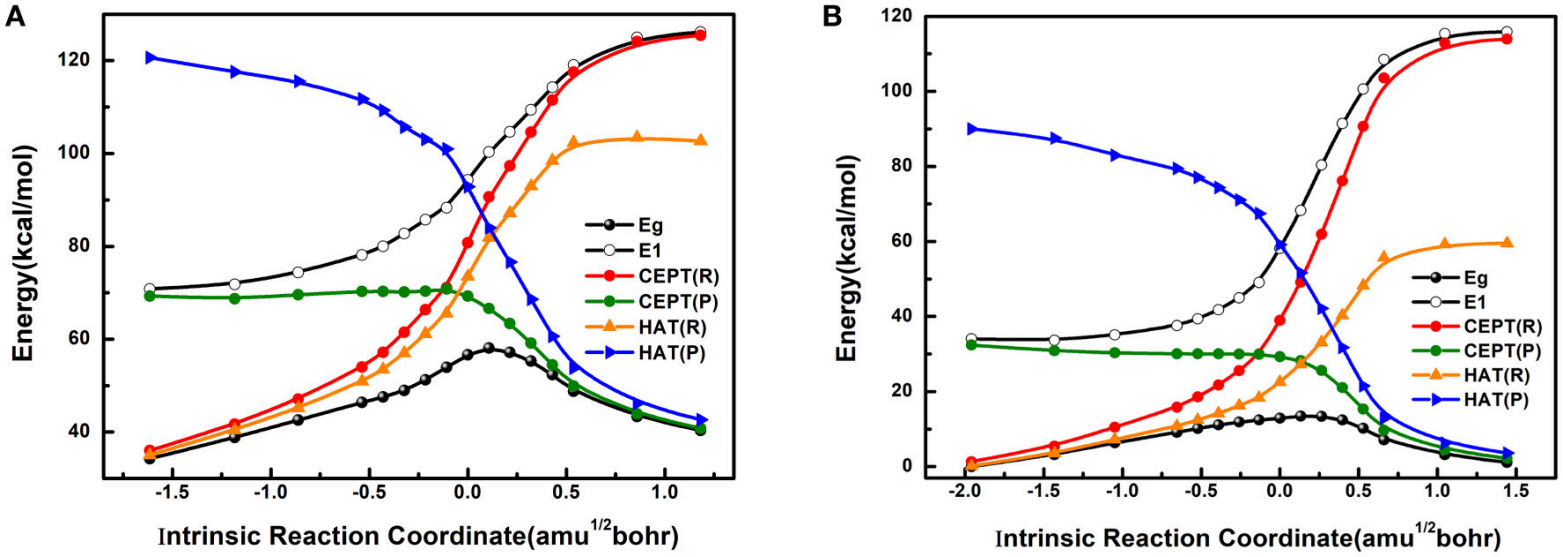
The effective diabatic and adiabatic potential energy surfaces for triplet **(A)** and quintet **(B)** high-valent oxoiron (IV) complexes along IRC in the C-H bond activation reactions. CEPT(R) (red line) and CEPT(P) (green line) represent the effective reactant state and product state of CEPT reaction mechanism, HAT(R) (orange line) and HAT(P) (blue line) mean the effective reactant state and product state of HAT reaction mechanism. The two black lines stand for the ground state and excited state. Use the lowest energy of the quintet ground state as the zero-point energy. The abscissa and ordinate stand for reaction coordinate from IRC (amu^1/2^bohr) and energy (kcal mol^−1^), respectively.

The computed potential energy profiles for the reactant (R) and product (P) diabatic states corresponding to the HAT and CEPT reaction mechanisms are shown in Figure 4 (more data are shown in Supplementary Material), both in the triplet (Figure 4A) and quintet (Figure 4B) spin states. Figure 4 also displays the potential energy curves of the adiabatic ground and excited state determined by MSDFT, along the intrinsic reaction coordinate obtained from KS-DFT calculations. In general, the diabatic states that best match the adiabatic potential energy curves, reflected by the smaller energy gap between the crossing point of the diabatic states and the barrier on the adiabatic surface, can be denoted as the overall reaction mechanism. Thus, this procedure provides a straightforward way to identify the reaction mechanism, consistent with a VB state-interaction perspective.

Figure 4 shows that the reactions both in the triplet (Figure 4A) and quintet (Figure 4B) states follow the same reaction mechanism, which is a concerted proton and electron transfer, but it can neither be fully described by a HAT nor by a CEPT process throughout the reaction path. On the product side, the concerted electron-proton transfer, or CEPT diabatic state best resembles the reaction profile, which is asymptotically transformed into of the adiabatic ground state. Conversely, the reactant diabatic state of the CEPT(R) mechanism approaches the adiabatic excited state. On the other hand, the reactant diabatic states of the HAT mechanism is lower in energy than that of the CEPT mechanism, thereby, having a closer match to the adiabatic ground state. The difference is particularly striking in the quintet spin state in comparison with that of the triplet state (Figure 4). Since the maximum on the adiabatic ground state potential (TS) lags behind the diabatic crossing points either between the two CEPT states or between the HAT reactant state and the CEPT product state (as shown in Figure 4), the HAT reactant state has significant contributions to the adiabatic ground state prior to the crossing point, after which the dominant character switches to the CEPT product state. For comparison, according to the classic VB-correlation diagram developed by Shaik et al. (2008), Lai et al. (2012), and Usharani et al. (2013, 2014), the C-H bond activation by the reactive agent Fe^IV^(O)(N4Py)(ClO_4_)_2_ would have been best described as a concerted CEPT process for both triplet and quintet states. Because the diabatic crossing point for the HAT reaction mechanism occurs at a much higher energy (83.0 kcal mol^−1^ for triplet state and 36.8 kcal mol^−1^ for the quintet state) than the CEPT mechanism (71.0 kcal mol^−1^ for triplet state and 29.9 kcal mol^−1^ for the quintet state), after the CEPT crossing point the dominant VB character switches from HAT on the reactant side to purely CEPT on the product state. The energetic features of the HAT states prior to the diabatic crossing points, and the dominantly CEPT character afterwards are, in fact, fully consistent with the intuitive perspective of C-H activation reaction. Here, both the transferring electron and proton originate from an identical site in the reactant state, but they end up in different locations (as a proton and an electron, separately) in the product.

## Conclusions

In summary, both Kohn-Sham DFT and MSDFT calculations have been performed in the present study to investigate the mechanism of C-H activation, which transpires in the catalytic oxidation of N,N-dimethyalinines by the reactive species Fe^IV^(O)(N4Py)(ClO_4_)_2_. Computational results show that the overall reaction comprises of two processes: the initial C-H activation is followed by a barrierless hydroxyl radical recombination. Kohn-Sham DFT calculations reveal that the C-H bond activation occurs via a HAT mechanism, in accord with the recent predictions of PCET reactivity in analogous N-H and O-H bond activation reactions. In addition, the MSDFT method has been used to explore the diabatic and adiabatic potential energy surface along the reaction coordinate. Interestingly, the MSDFT calculations suggest that the mechanism involves an initial HAT mechanism prior to reaching the diabatic crossing point, after which the mechanism is dominated by the CEPT product formation. The present study offers a clear theoretical example of a concerted electron-proton transfer reaction in C-H bond activation by Fe^IV^(O)(N4Py)(ClO_4_)_2_, and elucidates the explicit pathway for N-dealkylation of N,N-dimethylaniline mediated by nonheme oxo-iron(IV) complexes in drug metabolism. More importantly, the present study shows that MSDFT approach can be used to investigate other hydrogen abstraction process in a diabatic point of view.

## Author contributions

All authors listed have made a substantial, direct and intellectual contribution to the work, and approved it for publication.

### Conflict of interest statement

The authors declare that the research was conducted in the absence of any commercial or financial relationships that could be construed as a potential conflict of interest.
