# Supplementary material for: Combined Multistate and Kohn-Sham Density Functional Theory Studies of the Elusive Mechanism of N-Dealkylation of N,N-Dimethylanilines Mediated by the Biomimetic Nonheme Oxidant FeIV(O)(N4Py)(ClO4)2
Source: Front Chem. 2018 Sep 10;6:406. doi: 10.3389/fchem.2018.00406 (PMC6139341; doi:10.3389/fchem.2018.00406)
Supplement: Supplementary file 1 [file Table_1.doc]

Supplementary Material

**Combined Multistate and Kohn-Sham Density Functional Theory Studies of the elusive mechanism of *N*-dealkylation of *N,N*-dimethylanilines Mediated by the** **biomimetic nonheme oxidant Fe^IV^(O)(N4Py)(ClO_4_)_2_**

Lili Yang, Xin Chen, Zexing Qu^*^, Jiali Gao

*** Correspondence:** Zexing Qu: zxqu@jlu.edu.cn

## Supplementary Figures


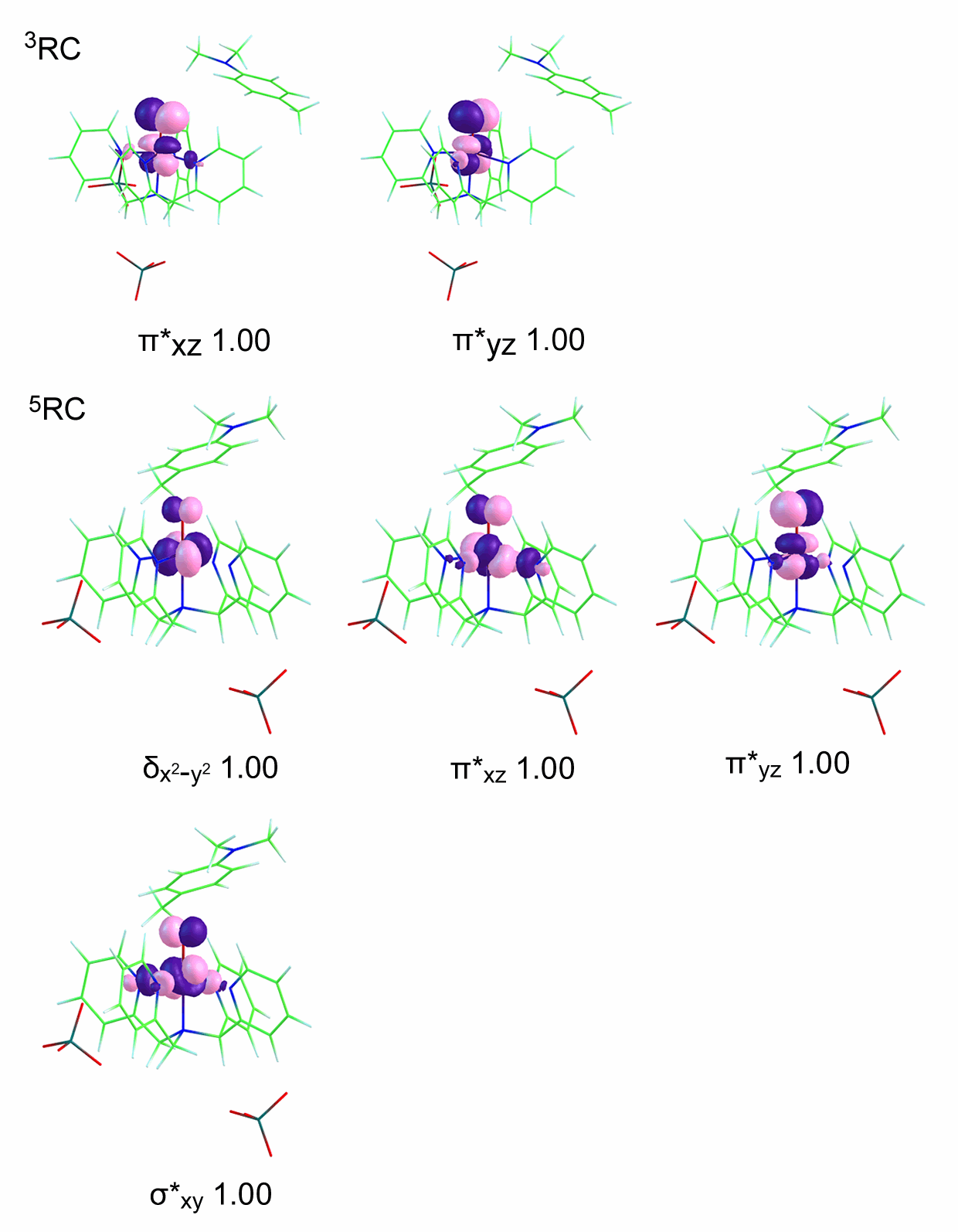

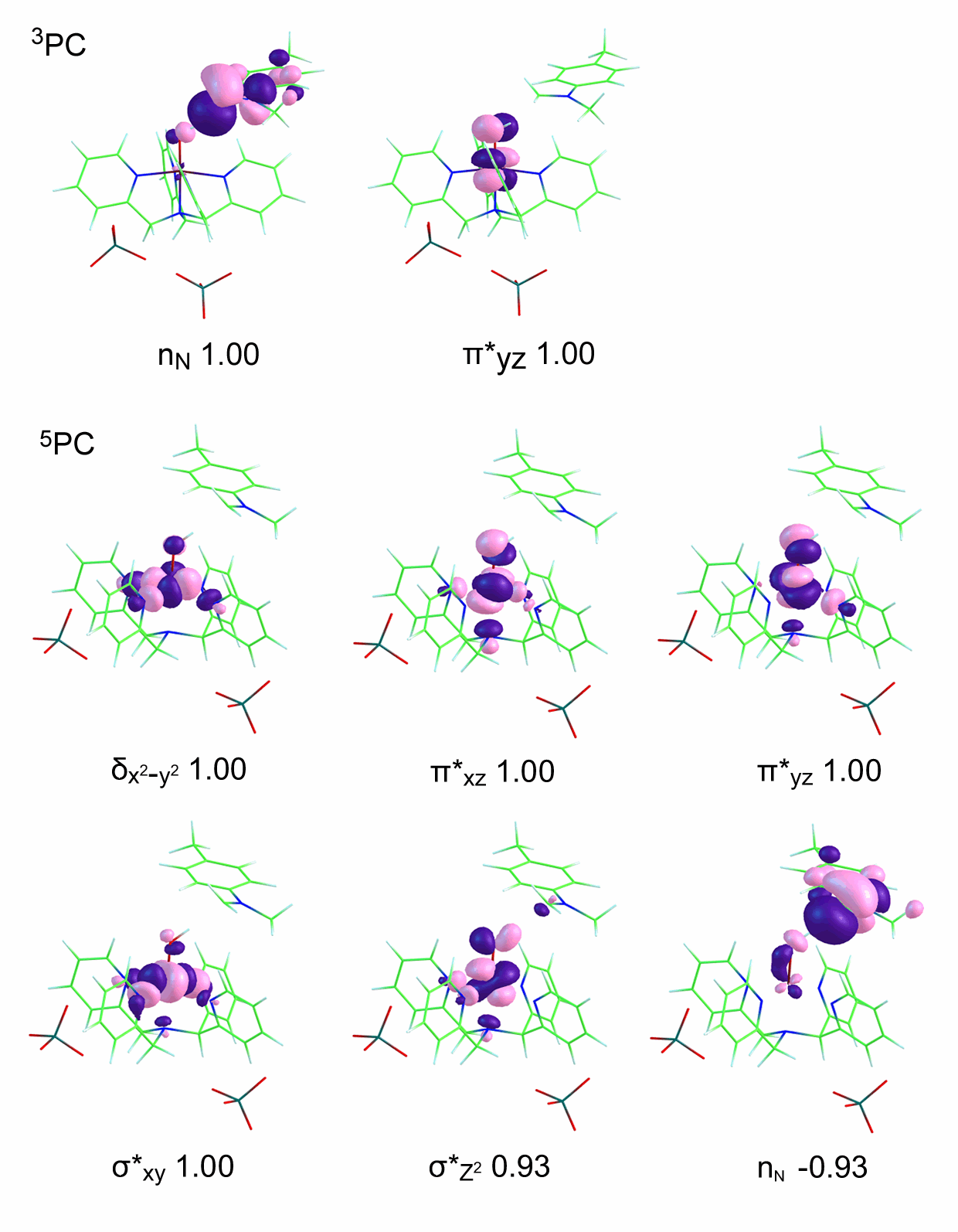


**Supplementary Figure 1.** Spin natural orbitals (SNO) and their occupation numbers of reactant and product in the H-transfer reaction of para-substituted N,N-dimethylanilines mediated by [(N4Py)-Fe^IV^=O] (ClO_4_)_2_. A negative occupation number corresponds to spin β.


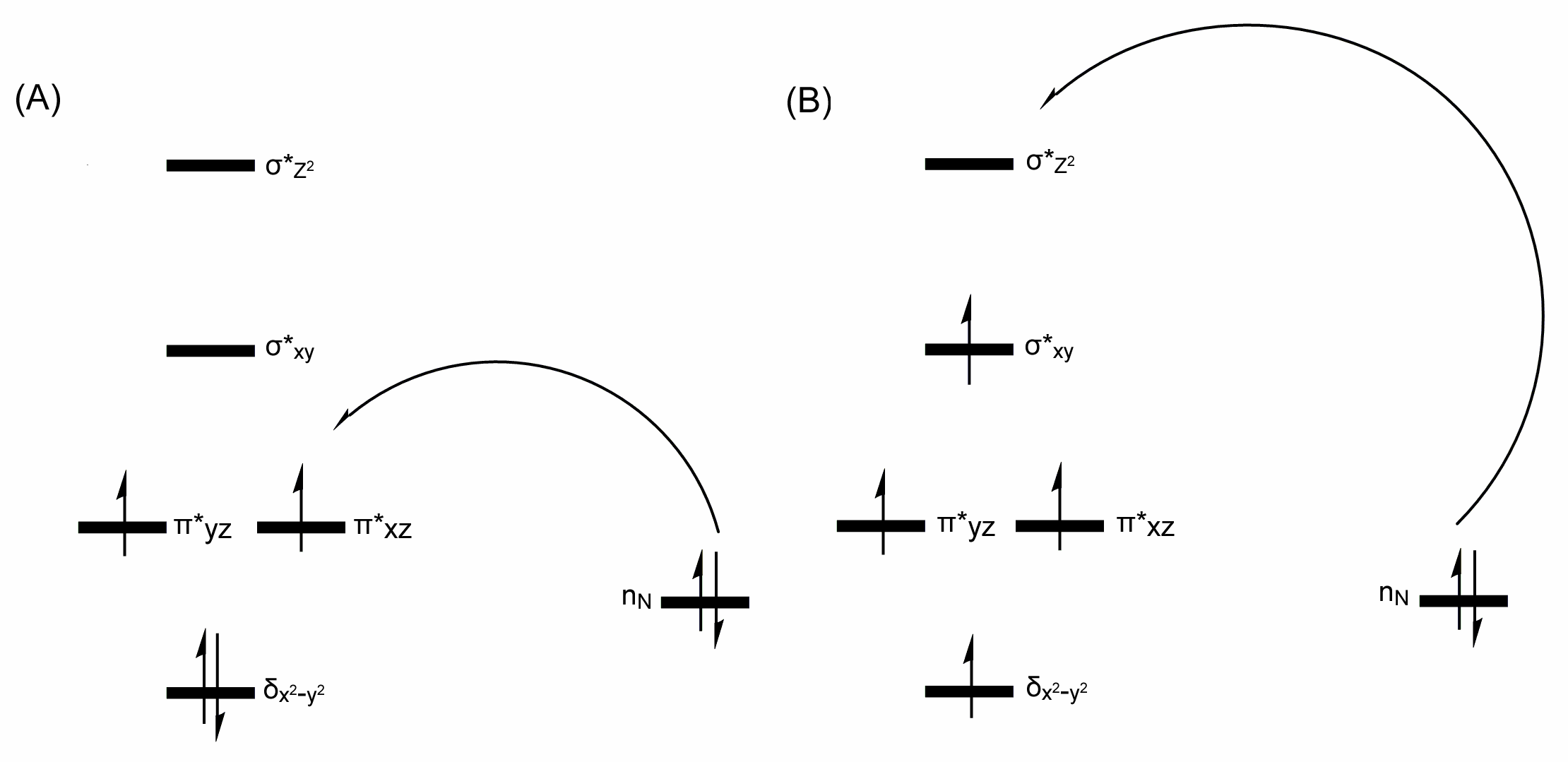


**Supplementary Figure 2.** The schematic diagram of electron transfer during the C-H bond activation of para-substituted N,N-dimethylanilines mediated by [(N4Py)-Fe^IV^=O](ClO_4_)_2_ for (A)triplet and (B)quintet pathways.


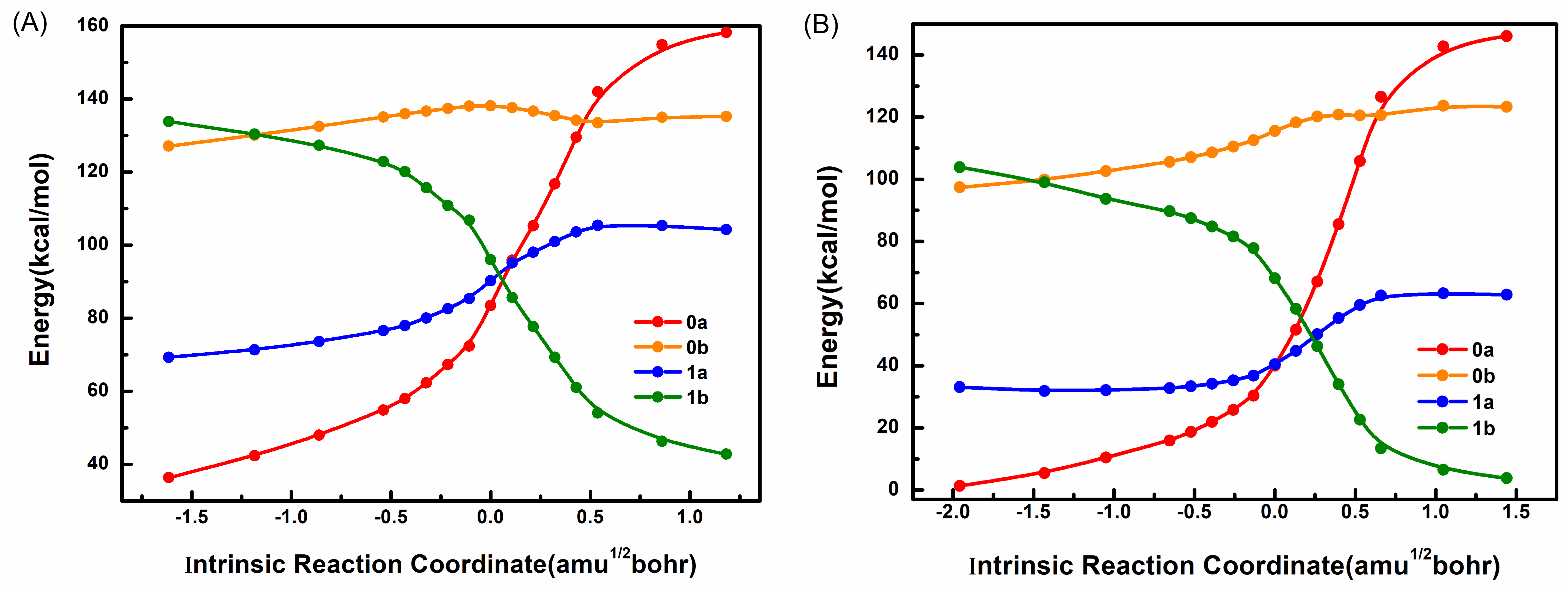


**Supplementary Figure 3.** Diabatic potential energy surfaces for the triplet(A) and quintet(B) high-valent oxoiron (IV) complexes along IRC in the C-H bond activation reactions. 0a (red line), 0b (orange line), 1a (blue line) and 1b (green line) represent for reactant state, proton transfer state, electron transfer state and product state respectively. Use the lowest energy of the quintet ground state as the zero-point energy. The abscissa and ordinate stand for reaction coordinate from IRC (amu^1/2^bohr) and energy (kcal mol^-1^) respectively.


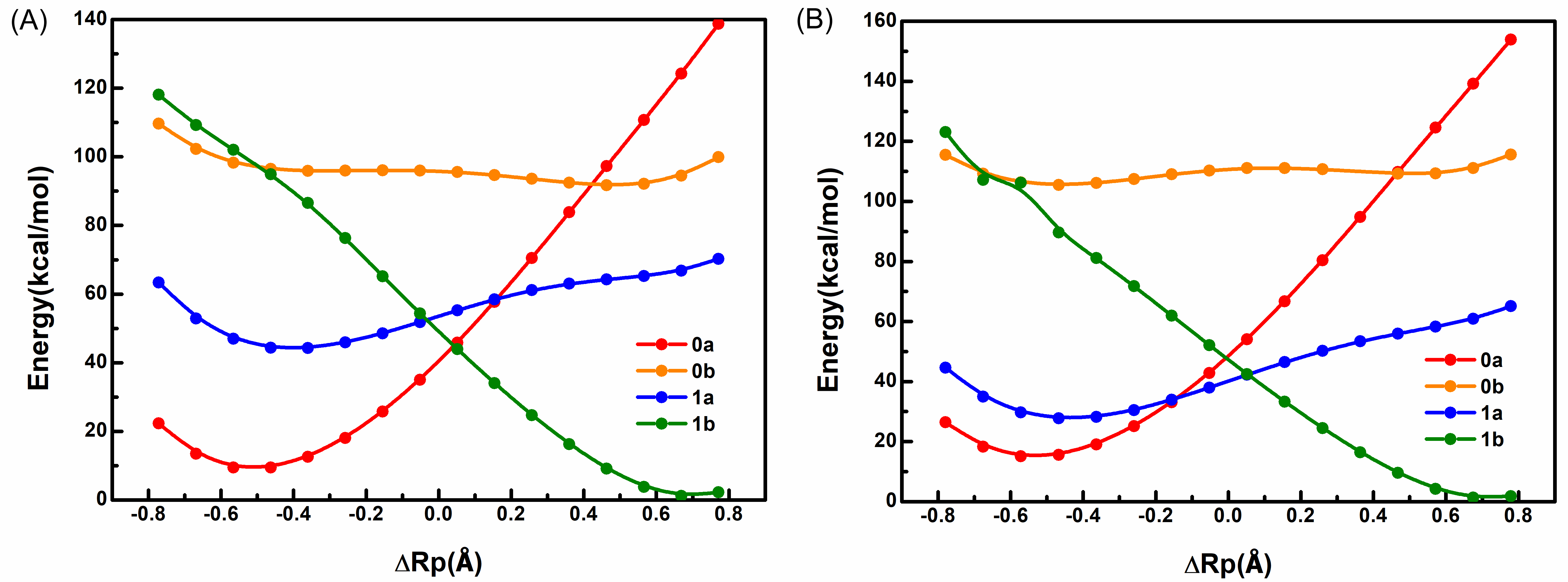


**Supplementary Figure 4.** Diabatic potential energy surfaces for the triplet(A) and quintet(B) high-valent oxoiron (IV) complexes along ΔRp in the C-H bond activation reactions. 0a (red line), 0b (orange line), 1a (blue line) and 1b (green line) represent for reactant state, proton transfer state, electron transfer state and product state respectively. Use the lowest energy of the quintet ground state as the zero-point energy. The abscissa and ordinate stand for the distance of the migrating hydrogen from the donor atom C and from the acceptor atom O (Å) in C-H bond activation step and energy (kcal mol^-1^) respectively.


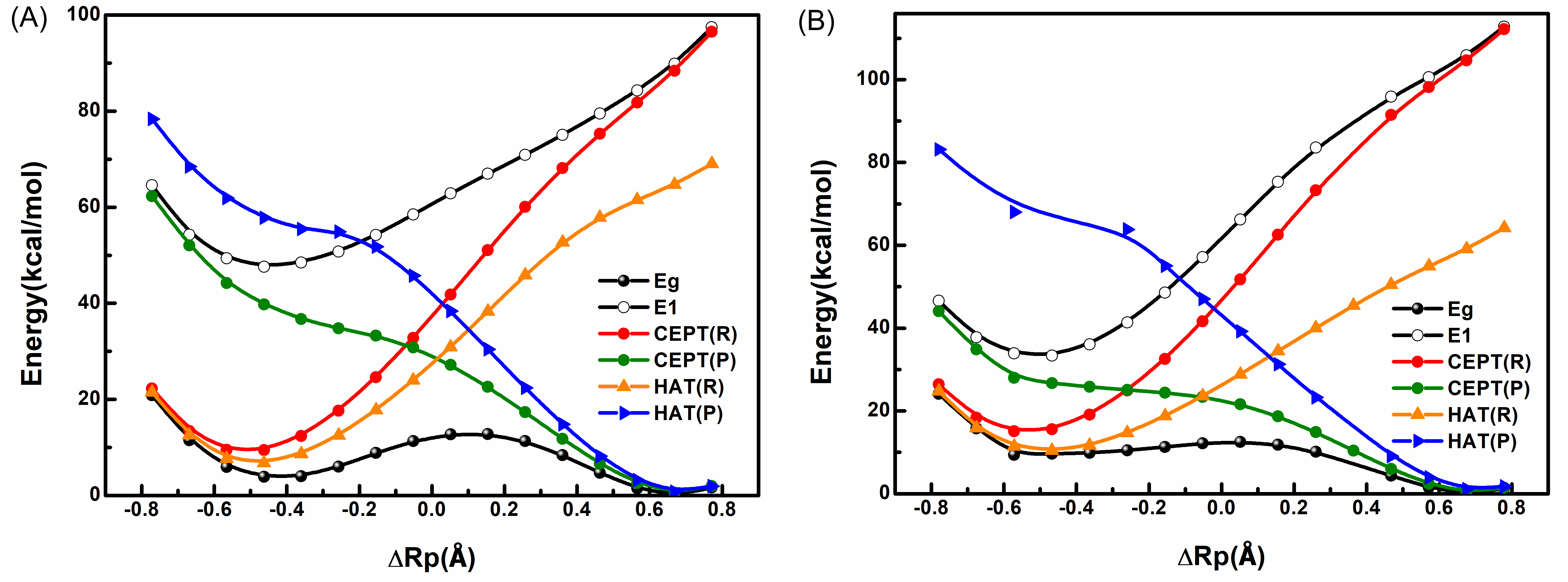


**Supplementary Figure 5.**  The effective diabatic and adiabatic potential energy surfaces for the triplet(A) and quintet(B) high-valent oxoiron (IV) complexes along ΔRp in the C-H bond activation reactions. CEPT(R) (red line) and CEPT(P) (green line) represent the effective reactant state and product state of CEPT reaction mechanism, HAT(R) (orange line) and HAT(P) (blue line) mean the effective reactant state and product state of HAT reaction mechanism. The two black lines stand for the ground state and excited state. Use the lowest energy of the quintet ground state as the zero-point energy. The abscissa and ordinate stand for the distance of the migrating hydrogen from the donor atom C and from the acceptor atom O (Å) in C-H bond activation step and energy (kcal mol^-1^) respectively.

## Supplementary Tables

**Supplementary Table 1.** Energy of diabatic states for triplet high-valent oxoiron (IV) complexes along the intrinstic reaction coordinate(IRC) in C-H bond activation step.

| IRC | ^3^RC | ^3^PT | ^3^ET | ^3^PC |
| --- | --- | --- | --- | --- |
| -1.61524 | -4427.209 | -4427.06443 | -4427.1565 | -4427.0539 |
| -1.18425 | -4427.1994 | -4427.05967 | -4427.15322 | -4427.0591 |
| -0.86095 | -4427.1905 | -4427.05574 | -4427.1497 | -4427.064 |
| -0.53781 | -4427.1795 | -4427.05169 | -4427.1449 | -4427.07116 |
| -0.43019 | -4427.1746 | -4427.05036 | -4427.1427 | -4427.0755 |
| -0.32259 | -4427.1677 | -4427.04911 | -4427.1394 | -4427.0825 |
| -0.215 | -4427.1597 | -4427.04801 | -4427.1353 | -4427.09027 |
| -0.1078 | -4427.15161 | -4427.04691 | -4427.1309 | -4427.09675 |
| 0 | -4427.13399 | -4427.04681 | -4427.1231 | -4427.1139 |
| 0.1078 | -4427.11415 | -4427.04761 | -4427.1153 | -4427.13044 |
| 0.21386 | -4427.09912 | -4427.04916 | -4427.1107 | -4427.14308 |
| 0.32141 | -4427.08092 | -4427.05111 | -4427.106 | -4427.15651 |
| 0.42908 | -4427.06057 | -4427.05308 | -4427.1018 | -4427.16964 |
| 0.53681 | -4427.04062 | -4427.05421 | -4427.0989 | -4427.18087 |
| 0.85928 | -4427.02007 | -4427.05182 | -4427.0991 | -4427.19314 |
| 1.1822 | -4427.01474 | -4427.05144 | -4427.1008 | -4427.19878 |

**Supplementary Table 2.** Energy of diabatic states for quintet high-valent oxoiron (IV) complexes along the intrinstic reaction coordinate(IRC) in C-H bond activation step.

| IRC | ^5^RC | ^5^PT | ^5^ET | ^5^PC |
| --- | --- | --- | --- | --- |
| -1.9593 | -4427.2648 | -4427.1117 | -4427.214 | -4427.1014 |
| -1.4312 | -4427.2582 | -4427.1079 | -4427.216 | -4427.1091 |
| -1.04921 | -4427.2502 | -4427.1034 | -4427.216 | -4427.1177 |
| -0.65395 | -4427.2416 | -4427.0987 | -4427.215 | -4427.1239 |
| -0.52184 | -4427.2372 | -4427.0963 | -4427.214 | -4427.1276 |
| -0.38988 | -4427.232 | -4427.0938 | -4427.213 | -4427.1318 |
| -0.25858 | -4427.2258 | -4427.0908 | -4427.211 | -4427.137 |
| -0.13218 | -4427.2186 | -4427.0876 | -4427.208 | -4427.1429 |
| 0 | -4427.2032 | -4427.0828 | -4427.202 | -4427.1583 |
| 0.13218 | -4427.1848 | -4427.0785 | -4427.196 | -4427.1741 |
| 0.26413 | -4427.1601 | -4427.0755 | -4427.187 | -4427.1932 |
| 0.39627 | -4427.1307 | -4427.0745 | -4427.179 | -4427.2128 |
| 0.52841 | -4427.0983 | -4427.0749 | -4427.172 | -4427.2308 |
| 0.66055 | -4427.0653 | -4427.0748 | -4427.167 | -4427.2455 |
| 1.04704 | -4427.0394 | -4427.0699 | -4427.166 | -4427.2565 |
| 1.4422 | -4427.0341 | -4427.0705 | -4427.167 | -4427.2609 |

**Supplementary Table 3.** Energy of the effective diabatic and adiabatic states for triplet high-valent oxoiron (IV) complexes along the intrinstic reaction coordinate(IRC) in C-H bond activation step.

| IRC | CEPT(R) | CEPT(P) | HAT(R) | HAT(P) | Eg | E1 |
| --- | --- | --- | --- | --- | --- | --- |
| -1.61524 | -4427.20953 | -4427.15649 | -4427.21107 | -4427.07467 | -4427.21231 | -4427.15408 |
| -1.18425 | -4427.2005 | -4427.15743 | -4427.20249 | -4427.07959 | -4427.20507 | -4427.15246 |
| -0.86095 | -4427.19188 | -4427.156 | -4427.19493 | -4427.08282 | -4427.19907 | -4427.14842 |
| -0.53781 | -4427.18087 | -4427.15491 | -4427.18576 | -4427.08886 | -4427.19305 | -4427.14247 |
| -0.43019 | -4427.17587 | -4427.1549 | -4427.18173 | -4427.09277 | -4427.19118 | -4427.1394 |
| -0.32259 | -4427.16901 | -4427.15515 | -4427.17609 | -4427.09851 | -4427.18894 | -4427.13506 |
| -0.215 | -4427.16119 | -4427.15482 | -4427.16944 | -4427.10273 | -4427.18531 | -4427.13034 |
| -0.1078 | -4427.15362 | -4427.1541 | -4427.16246 | -4427.10604 | -4427.18098 | -4427.12617 |
| 0 | -4427.13826 | -4427.15648 | -4427.14972 | -4427.11898 | -4427.17666 | -4427.11665 |
| 0.1078 | -4427.12245 | -4427.16076 | -4427.13637 | -4427.13308 | -4427.17437 | -4427.10705 |
| 0.21386 | -4427.1118 | -4427.16601 | -4427.12802 | -4427.14482 | -4427.17583 | -4427.10016 |
| 0.32141 | -4427.10031 | -4427.17266 | -4427.11874 | -4427.15758 | -4427.17884 | -4427.09263 |
| 0.42908 | -4427.08923 | -4427.18018 | -4427.10999 | -4427.17027 | -4427.18355 | -4427.08485 |
| 0.53681 | -4427.07962 | -4427.18743 | -4427.10387 | -4427.18125 | -4427.18921 | -4427.07719 |
| 0.85928 | -4427.06918 | -4427.19697 | -4427.10208 | -4427.19336 | -4427.19789 | -4427.06781 |
| 1.1822 | -4427.06705 | -4427.20191 | -4427.10332 | -4427.19897 | -4427.20264 | -4427.06591 |

**Supplementary Table 4.** Energy of the effective diabatic and adiabatic states for quintet high-valent oxoiron (IV) complexes along the intrinstic reaction coordinate(IRC) in C-H bond activation step.

| IRC | CEPT(R) | CEPT(P) | HAT(R) | HAT(P) | Eg | E1 |
| --- | --- | --- | --- | --- | --- | --- |
| -1.9593 | -4427.26484 | -4427.21532 | -4427.26666 | -4427.1235 | -4427.26693 | -4427.21268 |
| -1.4312 | -4427.25822 | -4427.21773 | -4427.2613 | -4427.12746 | -4427.26188 | -4427.21333 |
| -1.04921 | -4427.25019 | -4427.21865 | -4427.25537 | -4427.13471 | -4427.25679 | -4427.21103 |
| -0.65395 | -4427.24171 | -4427.21901 | -4427.24973 | -4427.14033 | -4427.25236 | -4427.20705 |
| -0.52184 | -4427.23732 | -4427.21907 | -4427.2471 | -4427.14408 | -4427.25071 | -4427.20417 |
| -0.38988 | -4427.23228 | -4427.21908 | -4427.24427 | -4427.14836 | -4427.24925 | -4427.20036 |
| -0.25858 | -4427.22618 | -4427.21918 | -4427.24107 | -4427.15366 | -4427.24803 | -4427.19523 |
| -0.13218 | -4427.21921 | -4427.21931 | -4427.23768 | -4427.1594 | -4427.24714 | -4427.18876 |
| 0 | -4427.20484 | -4427.2203 | -4427.23096 | -4427.17258 | -4427.24639 | -4427.17429 |
| 0.13218 | -4427.1886 | -4427.22193 | -4427.22349 | -4427.1846 | -4427.24542 | -4427.15812 |
| 0.26413 | -4427.16827 | -4427.22614 | -4427.21416 | -4427.19973 | -4427.24555 | -4427.1389 |
| 0.39627 | -4427.14559 | -4427.23338 | -4427.20274 | -4427.21637 | -4427.24718 | -4427.12126 |
| 0.52841 | -4427.12246 | -4427.24247 | -4427.19007 | -4427.23261 | -4427.25067 | -4427.10664 |
| 0.66055 | -4427.10193 | -4427.25148 | -4427.1781 | -4427.24638 | -4427.2556 | -4427.09411 |
| 1.04704 | -4427.08692 | -4427.25975 | -4427.17255 | -4427.25693 | -4427.2619 | -4427.08294 |
| 1.4422 | -4427.08529 | -4427.26347 | -4427.17204 | -4427.26122 | -4427.26514 | -4427.08222 |

**Supplementary Table 5.** Energy of diabatic states for triplet high-valent oxoiron (IV) complexes along the distance of the migrating hydrogen from the donor atom C and from the acceptor atom O (ΔRp)in C-H bond activation step.

| ΔRp | ^3^RC | ^3^PT | ^3^ET | ^3^PC |
| --- | --- | --- | --- | --- |
| -0.8744 | -4427.1916 | -4427.0574 | -4427.124 | -4427.0433 |
| -0.77152 | -4427.216 | -4427.0768 | -4427.1505 | -4427.0633 |
| -0.66866 | -4427.2301 | -4427.0885 | -4427.1672 | -4427.0774 |
| -0.56578 | -4427.2364 | -4427.0949 | -4427.1767 | -4427.0889 |
| -0.46292 | -4427.2364 | -4427.0978 | -4427.1808 | -4427.1003 |
| -0.36004 | -4427.2315 | -4427.0987 | -4427.181 | -4427.1136 |
| -0.25718 | -4427.2227 | -4427.0986 | -4427.1784 | -4427.1299 |
| -0.1543 | -4427.2105 | -4427.0984 | -4427.1741 | -4427.1476 |
| -0.05144 | -4427.1957 | -4427.0985 | -4427.1689 | -4427.1649 |
| 0.05144 | -4427.1785 | -4427.0992 | -4427.1634 | -4427.1815 |
| 0.1543 | -4427.1595 | -4427.1006 | -4427.1583 | -4427.1973 |
| 0.25718 | -4427.1391 | -4427.1024 | -4427.1541 | -4427.2122 |
| 0.36004 | -4427.1179 | -4427.1042 | -4427.1511 | -4427.2256 |
| 0.46292 | -4427.0965 | -4427.1053 | -4427.1491 | -4427.237 |
| 0.56578 | -4427.0751 | -4427.1047 | -4427.1475 | -4427.2454 |
| 0.66866 | -4427.0535 | -4427.1009 | -4427.145 | -4427.2496 |
| 0.77152 | -4427.0304 | -4427.0923 | -4427.1395 | -4427.248 |
| 0.8744 | -4427.0033 | -4427.0763 | -4427.1281 | -4427.238 |

**Supplementary Table 6.** Energy of diabatic states for quintet high-valent oxoiron (IV) complexes along the distance of the migrating hydrogen from the donor atom C and from the acceptor atom O (ΔRp) in C-H bond activation step.

| ΔRp | RC | PT | ET | PC |
| --- | --- | --- | --- | --- |
| -0.88324 | -4427.1911 |  | -4427.16 | -4427.0409 |
| -0.77932 | -4427.214 | -4427.072 | -4427.185 | -4427.06 |
| -0.67542 | -4427.2269 | -4427.082 | -4427.2004 | -4427.0854 |
| -0.5715 | -4427.2321 | -4427.0867 | -4427.2088 | -4427.0868 |
| -0.4676 | -4427.2313 | -4427.088 | -4427.2119 | -4427.1133 |
| -0.36368 | -4427.2257 | -4427.087 | -4427.2111 | -4427.1268 |
| -0.25978 | -4427.2161 | -4427.0849 | -4427.2075 | -4427.1417 |
| -0.15586 | -4427.2034 | -4427.0824 | -4427.2021 | -4427.1574 |
| -0.05196 | -4427.1878 | -4427.0804 | -4427.1956 | -4427.1731 |
| 0.05196 | -4427.1699 | -4427.0791 | -4427.1888 | -4427.1884 |
| 0.15586 | -4427.1498 | -4427.079 | -4427.1821 | -4427.2031 |
| 0.25978 | -4427.128 | -4427.0797 | -4427.1761 | -4427.2171 |
| 0.36368 | -4427.105 | -- | -4427.171 | -4427.2299 |
| 0.4676 | -4427.0812 | -4427.082 | -4427.1669 | -4427.2409 |
| 0.5715 | -4427.0576 | -4427.0819 | -4427.1633 | -4427.2494 |
| 0.67542 | -4427.0343 | -4427.0791 | -4427.1591 | -4427.254 |
| 0.77932 | -4427.0108 | -4427.0719 | -4427.1524 | -4427.2532 |

**Supplementary Table 7.** Energy of the effective diabatic and adiabatic states for triplet high-valent oxoiron (IV) complexes along the distance of the migrating hydrogen from the donor atom C and from the acceptor atom O (ΔRp) in C-H bond activation step.

| ΔRp | CEPT(R) | CEPT(P) | HAT(R) | HAT(P) | Eg | E1 |
| --- | --- | --- | --- | --- | --- | --- |
| -0.8744 | -4427.1916 | -4427.1252 | -4427.1925 | -4427.1035 | -4427.1932 | -4427.1227 |
| -0.77152 | -4427.216 | -4427.1522 | -4427.2173 | -4427.1266 | -4427.2183 | -4427.1486 |
| -0.66866 | -4427.2301 | -4427.1686 | -4427.2315 | -4427.1424 | -4427.2332 | -4427.165 |
| -0.56578 | -4427.2364 | -4427.1811 | -4427.2393 | -4427.1528 | -4427.2421 | -4427.1728 |
| -0.46292 | -4427.2365 | -4427.1881 | -4427.2407 | -4427.1594 | -4427.2453 | -4427.1756 |
| -0.36004 | -4427.2318 | -4427.193 | -4427.2377 | -4427.1631 | -4427.2451 | -4427.1742 |
| -0.25718 | -4427.2234 | -4427.1961 | -4427.2316 | -4427.164 | -4427.242 | -4427.1706 |
| -0.1543 | -4427.2123 | -4427.1985 | -4427.2232 | -4427.169 | -4427.2374 | -4427.1651 |
| -0.05144 | -4427.1992 | -4427.2024 | -4427.2133 | -4427.1786 | -4427.2335 | -4427.1583 |
| 0.05144 | -4427.1849 | -4427.2082 | -4427.2023 | -4427.1903 | -4427.2312 | -4427.1513 |
| 0.1543 | -4427.1701 | -4427.2155 | -4427.1904 | -4427.203 | -4427.2311 | -4427.1448 |
| 0.25718 | -4427.1558 | -4427.2239 | -4427.1784 | -4427.2158 | -4427.2335 | -4427.1385 |
| 0.36004 | -4427.1429 | -4427.2327 | -4427.1675 | -4427.2279 | -4427.2381 | -4427.1319 |
| 0.46292 | -4427.1315 | -4427.241 | -4427.1593 | -4427.2385 | -4427.244 | -4427.1248 |
| 0.56578 | -4427.1212 | -4427.2474 | -4427.1534 | -4427.2464 | -4427.2491 | -4427.1172 |
| 0.66866 | -4427.1106 | -4427.2506 | -4427.1484 | -4427.2503 | -4427.2515 | -4427.1083 |
| 0.77152 | -4427.0977 | -4427.2484 | -4427.1415 | -4427.2484 | -4427.2489 | -4427.0962 |

**Supplementary Table 8. Energy of the effective diabatic and adiabatic states for quintet high-valent oxoiron (IV) complexes along the distance of the migrating hydrogen from the donor atom C and from the acceptor atom O (ΔRp) in C-H bond activation step.**

|  | CEPT(R) | CEPT(P) | HAT(R) | HAT(P) | Eg | E1 |
| --- | --- | --- | --- | --- | --- | --- |
| -0.88324 |  | -4427.1606 | -4427.1929 |  |  |  |
| -0.77932 | -4427.214 | -4427.1859 | -4427.2166 | -4427.1236 | -4427.2177 | -4427.1818 |
| -0.67542 | -4427.2269 | -4427.2006 | -4427.2308 | -- | -4427.231 | -4427.1959 |
| -0.5715 | -4427.2321 | -4427.2115 | -4427.2378 | -4427.1477 | -4427.2411 | -4427.2021 |
| -0.4676 | -4427.2313 | -4427.2135 | -4427.2395 | -- | -4427.2407 | -4427.2029 |
| -0.36368 | -4427.2257 | -4427.215 | -4427.2375 | -- | -4427.2404 | -4427.1986 |
| -0.25978 | -4427.2164 | -4427.2161 | -4427.2327 | -4427.1544 | -4427.2394 | -4427.1902 |
| -0.15586 | -4427.2042 | -4427.2172 | -4427.2262 | -4427.1684 | -4427.2381 | -4427.1787 |
| -0.05196 | -4427.1897 | -4427.2188 | -4427.2185 | -4427.1811 | -4427.2367 | -4427.1651 |
| 0.05196 | -4427.1736 | -4427.2217 | -4427.2101 | -4427.1936 | -4427.2362 | -4427.1506 |
| 0.15586 | -4427.1564 | -4427.2263 | -4427.2012 | -4427.2063 | -4427.2372 | -4427.1361 |
| 0.25978 | -4427.1393 | -4427.2324 | -4427.1923 | -4427.219 | -4427.2399 | -4427.1229 |
| 0.36368 | -- | -4427.2395 | -4427.1836 | -- | -- | -- |
| 0.4676 | -4427.1103 | -4427.2465 | -4427.1756 | -4427.2416 | -4427.2492 | -4427.1033 |
| 0.5715 | -4427.0996 | -4427.2523 | -4427.1685 | -4427.2498 | -4427.2537 | -4427.0958 |
| 0.67542 | -4427.0893 | -4427.2555 | -4427.1619 | -4427.2543 | -4427.2561 | -4427.0874 |
| 0.77932 | -4427.0773 | -4427.2538 | -4427.1538 | -4427.2533 | -4427.2541 | -4427.0763 |

## Supplementary Cartesian Coordinates

**^3^RC**

Fe 0.145927 -0.019852 0.832843

N -1.436347 -0.400308 -0.439924

N -0.528829 -1.702652 1.690468

N 1.087379 -1.240548 -0.466824

N -1.206218 1.011832 1.826435

N 0.451277 1.507174 0.377096

O 1.398680 0.258858 1.838523

C -0.393540 -2.087001 2.966791

C -0.965700 -3.271286 3.420691

C -1.696528 -4.056074 2.526798

C -1.835308 -3.651965 1.197848

C -1.227621 -2.460595 0.817461

C -1.212032 -1.883406 -0.589016

C 0.208297 -2.056946 -1.087686

C 0.613823 -2.982607 -2.040680

C 1.975007 -3.058172 -2.347438

C 2.875226 -2.213839 -1.698024

C 2.393470 -1.306044 -0.756269

C -0.915266 1.838315 2.848972

C -1.896958 2.577637 3.486470

C -3.214744 2.473532 3.038129

C -3.506615 1.622587 1.980851

C -2.476318 0.895803 1.386279

C -2.728995 -0.050563 0.244321

C -1.243126 0.372727 -1.709627

C -0.321083 1.544949 -1.482288

C -0.244280 2.612897 -2.372816

C 0.641370 3.650772 -2.111796

C 1.440066 3.594121 0.969585

C 1.310071 2.509834 0.117094

H 0.186353 -1.428325 3.603201

H -0.841643 -3.564086 4.457224

H -2.165320 -4.975515 2.862529

H -2.417140 -4.206179 0.468500

H -1.936655 -2.357667 -1.248803

H -0.125754 -3.615962 -2.519199

H 2.325592 -3.768933 -3.089168

H 3.938990 -2.240535 -1.904508

H 3.046914 -0.624807 -0.224195

H 0.129904 1.883236 3.130257

H -1.625793 3.243169 4.297742

H -4.000790 3.069298 3.488910

H -4.509416 1.548285 1.577827

H -0.916027 2.649881 -3.221651

H 0.683671 4.507574 2.774902

H 2.128569 4.393251 0.720432

H 1.881482 2.409117 0.797394

H -3.412320 0.413194 -0.466859

H -3.220027 -0.968006 0.584142

H -2.203132 0.726759 -2.086483

H -0.817337 -0.290935 -2.468072

Cl -2.763509 4.066393 0.471120

O -2.404898 4.994005 -1.580745

O -3.129689 2.718241 -1.058377

O -3.914334 4.581674 0.318434

O -1.574233 3.880325 0.432169

Cl -4.360799 -3.101258 1.536251

O -4.568764 -2.796340 -0.076276

O -5.595702 -3.631277 -2.146500

O -3.238286 -4.115382 -1.646301

O -3.909093 -1.844082 -2.230692

N 5.644774 -0.834348 1.923691

C 5.770889 -0.334237 0.632680

C 5.970563 0.659867 2.051572

C 6.491566 -1.033307 -0.364801

C 5.173811 0.888308 0.249139

C 6.579903 -0.541704 -1.664330

C 5.274329 1.358346 1.060220

C 6.519061 -1.913915 2.352822

C 5.027452 -0.004248 2.947363

C 6.064853 1.170618 3.469846

H 6.999110 -1.959574 -0.124853

H 4.639494 1.486162 0.976862

H 7.154365 -1.107490 -2.394885

H 4.800467 2.305171 1.309268

H 7.586890 -1.655591 2.280859

H 6.351051 -2.820743 1.760442

H 6.297466 -2.159629 3.392186

H 5.585546 0.925452 3.142155

H 4.976803 -0.570148 3.878912

H 4.002015 0.259887 2.667333

H 5.397133 0.622608 4.146979

H 5.791522 2.227977 3.530648

H 7.079566 1.066098 3.868351

**^3^TS**

Fe -0.589828 0.953901 -0.504737

N -0.314380 0.416825 1.454331

N 1.409983 1.048962 -0.456719

N -0.370497 -1.012002 -0.774147

N -0.704497 2.790080 0.250049

N -2.512827 0.718015 -0.067404

O -0.876057 1.445735 -2.148295

C 2.221708 1.836615 -1.176341

C 3.595317 1.853946 -0.956184

C 4.124264 1.044848 0.050218

C 3.278089 0.239164 0.814929

C 1.917071 0.268192 0.525042

C 0.839953 -0.542460 1.234109

C 0.329427 -1.574304 0.237441

C 0.536368 -2.947378 0.317786

C -0.001610 -3.747412 -0.692620

C -0.725740 -3.161893 -1.731835

C -0.898671 -1.780541 -1.736699

C -1.083649 3.877279 -0.447759

C -1.189729 5.123488 0.150766

C -0.911372 5.238997 1.515643

C -0.529962 4.111021 2.230994

C -0.422544 2.889617 1.564103

C 0.038038 1.633459 2.262071

C -1.569256 -0.232692 1.964147

C -2.765008 0.229586 1.165069

C -4.069848 0.129190 1.648299

C -5.120954 0.512887 0.822971

C -4.848933 0.994079 -0.459919

C -3.526808 1.095335 -0.867151

H 1.739007 2.454510 -1.924444

H 4.229312 2.497083 -1.556673

H 5.190764 1.042892 0.251598

H 3.658222 -0.389542 1.617325

H 1.204550 -1.018815 2.150332

H 1.100715 -3.351335 1.151072

H 0.141912 -4.822777 -0.659815

H -1.156073 -3.755513 -2.530646

H -1.455712 -1.264761 -2.509279

H -1.292649 3.697068 -1.496355

H -1.498761 5.980152 -0.437613

H -1.009806 6.195013 2.019522

H -0.347051 4.154025 3.297890

H -4.239590 -0.177196 2.674607

H -6.141807 0.457398 1.186868

H -5.643954 1.307695 -1.127319

H -3.227657 1.479937 -1.835831

H -0.409199 1.571180 3.260250

H 1.124526 1.669822 2.388973

H -1.702832 -0.020533 3.029468

H -1.469045 -1.317085 1.858029

Cl -2.983984 1.949504 4.685050

O -4.151268 1.015860 4.713406

O -1.713572 1.149688 4.892133

O -3.104196 2.989418 5.731301

O -2.896415 2.593373 3.321872

Cl 3.460025 -2.925069 2.708215

O 4.078321 -1.649262 3.206476

O 4.154451 -4.102261 3.270754

O 3.526433 -2.931972 1.203346

O 1.999056 -2.936168 3.105303

N 0.598752 -0.570291 -4.596493

C -0.490958 -1.206375 -5.202080

C -2.702459 -2.488775 -6.456933

C -0.357152 -2.485362 -5.778488

C -1.759208 -0.589081 -5.246645

C -1.445137 -3.101945 -6.389838

C -2.830732 -1.224389 -5.865689

C 1.795397 -1.342068 -4.277249

C 0.532332 0.781956 -4.203703

C -3.865522 -3.151216 -7.153663

H 0.597680 -2.997001 -5.769081

H -1.914625 0.369278 -4.765453

H -1.306844 -4.085496 -6.831436

H -3.796366 -0.725198 -5.879281

H 2.358060 -1.599423 -5.183242

H 1.540129 -2.266994 -3.750986

H 2.440513 -0.747722 -3.630907

H -0.010909 1.402007 -4.921230

H 1.528743 1.183043 -4.019404

H -0.125233 0.999579 -3.122532

H -4.817658 -2.887251 -6.683580

H -3.927155 -2.842703 -8.204852

H -3.772886 -4.241005 -7.140868

**^3^IM**

Fe -0.028322 0.714073 -0.728704

N 0.277501 0.050308 1.155637

N 1.969379 0.815246 -0.743831

N 0.219721 -1.243965 -1.156402

N -0.120055 2.487566 0.134160

N -1.934861 0.443711 -0.305073

O -0.335516 1.332702 -2.389353

C 2.761370 1.621377 -1.465706

C 4.1427630 1.603219 -1.304824

C 4.700812 0.732254 -0.367082

C 3.875078 -0.098234 0.392263

C 2.502585 -0.020062 0.175075

C 1.441085 -0.868449 0.861713

C 0.955697 -1.840651 -0.192397

C 1.248400 -3.199225 -0.217029

C 0.749458 -3.957760 -1.278135

C -0.018644 -3.338056 -2.265582

C -0.269221 -1.970631 -2.170334

C -0.498358 3.617982 -0.491620

C -0.553394 4.830465 0.177695

C -0.216875 4.865567 1.534007

C 0.166627 3.693437 2.172470

C 0.213684 2.506273 1.440037

C 0.634305 1.198791 2.061445

C -0.960900 -0.662162 1.632339

C -2.170721 -0.180247 0.868507

C -3.471238 -0.378305 1.328836

C -4.532661 0.081063 0.556855

C -4.274968 0.711888 -0.662034

C -2.956999 0.883054 -1.060373

H 2.258518 2.272603 -2.171623

H 4.762835 2.263888 -1.900674

H 5.775328 0.705707 -0.216019

H 4.255339 -0.775610 1.150112

H 1.798829 -1.379303 1.753102

H 1.845527 -3.629178 0.580724

H 0.952632 -5.022876 -1.327898

H -0.423261 -3.894892 -3.103179

H -0.856614 -1.436235 -2.907878

H -0.744722 3.501116 -1.540814

H -0.866527 5.724068 -0.350370

H -0.271452 5.795214 2.091071

H 0.392885 3.673591 3.231610

H -3.626048 -0.812714 2.308874

H -5.548781 -0.029794 0.919637

H -5.078469 1.087735 -1.285643

H -2.669809 1.370609 -1.985066

H 0.125378 1.084867 3.019885

H 1.709536 1.176503 2.267615

H -1.117702 -0.511943 2.704573

H -0.830907 -1.736674 1.473660

Cl -2.978694 1.419791 4.192443

O -4.387593 1.457833 3.700720

O -2.536587 -0.019185 4.319516

O -2.846749 2.115451 5.492960

O -2.081728 2.084179 3.166231

Cl 3.292123 -1.521081 3.766087

O 3.541569 -0.067948 3.453121

O 3.998881 -1.924578 4.997359

O 3.777864 -2.340536 2.583888

O 1.809993 -1.737181 3.887625

N 1.142275 -0.830911 -5.330327

C -0.093439 -1.359217 -5.739900

C -2.599424 -2.419368 -6.580760

C -0.172358 -2.608443 -6.385468

C -1.293772 -0.656503 -5.505518

C -1.403611 -3.117525 -6.790785

C -2.511591 -1.184342 -5.924541

C 2.306181 -1.709069 -5.266319

C 1.289605 0.496221 -4.971835

C -3.923003 -2.963534 -7.060519

H 0.724436 -3.179575 -6.594982

H -1.278608 0.278255 -4.957075

H -1.429448 -4.081674 -7.292436

H -3.420101 -0.621869 -5.723887

H 2.696968 -1.935447 -6.266061

H 2.054342 -2.649221 -4.767391

H 3.091591 -1.214350 -4.694539

H 0.611772 1.208621 -5.427467

H 2.306339 0.817560 -4.784966

H 0.270935 0.961858 -3.063497

H -4.135696 -2.644847 -8.088702

H -3.933841 -4.057545 -7.052727

H -4.750437 -2.613551 -6.436160

**^3^PC**

Fe -0.231225 -0.321838 0.617210

N 1.649814 0.182756 -0.291908

N 0.302739 1.171841 1.860683

N -0.849362 1.226380 -0.744709

N 0.964247 -1.605820 1.716085

N -0.321043 -1.671521 -0.855493

O -2.351800 -0.762919 1.321300

C 0.003520 1.374181 3.154340

C 0.528321 2.436338 3.882266

C 1.407945 3.313005 3.246726

C 1.749111 3.096572 1.911793

C 1.179209 2.009022 1.251913

C 1.462051 1.667640 -0.208411

C 0.205951 2.031465 -0.992887

C 0.132561 3.116021 -1.865609

C -1.085134 3.364266 -2.499925

C -2.175473 2.530719 -2.248902

C -2.013558 1.467556 -1.365249

C 0.535050 -2.586453 2.530976

C 1.403701 -3.460741 3.164676

C 2.771761 -3.329226 2.923314

C 3.213484 -2.323301 2.074246

C 2.282137 -1.469365 1.482475

C 2.742483 -0.351851 0.559821

C 1.631882 -0.391425 -1.661265

C 0.646896 -1.539027 -1.790792

C 0.759333 -2.440436 -2.849305

C -0.131052 -3.500444 -2.948105

C -1.115695 -3.644340 -1.971574

C -1.162060 -2.720551 -0.940738

H -0.661111 0.649070 3.611913

H 0.257029 2.561624 4.924829

H 1.838438 4.150492 3.786341

H 2.445673 3.736331 1.380307

H 2.327975 2.220125 -0.577456

H 1.013099 3.727184 -2.035342

H -1.175978 4.199363 -3.187384

H -3.135386 2.691506 -2.727360

H -2.835025 0.795144 -1.139694

H -0.540901 -2.662881 2.657463

H 1.013966 -4.244841 3.803914

H 3.481237 -4.020959 3.364111

H 4.263186 -2.226199 1.823279

H 1.579297 -2.336716 -3.549869

H -0.029464 -4.226502 -3.746834

H -1.808843 -4.477600 -1.982372

H -1.870415 -2.832906 -0.126077

H 3.551347 -0.744240 -0.060693

H 3.159951 0.464413 1.158109

H 2.620178 -0.769963 -1.932947

H 1.375812 0.394219 -2.378391

Cl 2.960963 -4.277520 -0.851817

O 2.804469 -5.001702 -2.144794

O 3.446118 -2.869954 -1.134369

O 3.942237 -4.958704 0.036633

O 1.628151 -4.192599 -0.159626

Cl 3.659098 4.474996 -1.114341

O 4.224741 3.546917 -0.068948

O 4.650918 5.502903 -1.495727

O 2.415653 5.113917 -0.546940

O 3.255857 3.648682 -2.308452

N -4.197009 0.763045 1.760444

C -5.198888 0.427186 0.825100

C -7.247652 -0.228878 -1.058758

C -5.930114 1.429271 0.160446

C -5.508912 -0.913588 0.513288

C -6.928348 1.098262 -0.754003

C -6.511501 -1.222087 -0.403510

C -3.781384 2.157341 1.884497

C -3.348815 -0.249317 2.270923

C -8.349677 -0.575941 -2.030319

H -5.730893 2.475527 0.355293

H -4.990206 -1.737803 0.992536

H -7.471311 1.903334 -1.242724

H -6.720100 -2.268353 -0.612325

H -4.604222 2.774634 2.259657

H -3.436605 2.579192 0.932207

H -2.960343 2.221919 2.599106

H -3.930856 -1.104891 2.622320

H -2.761460 0.140937 3.098756

H -2.838040 -1.125497 0.566450

H -9.302599 -0.738059 -1.511527

H -8.508785 0.225203 -2.757849

H -8.123069 -1.492429 -2.583531

**^5^RC**

Fe 0.1865340 0.832144 -0.563936

N 0.3007020 0.024712 1.355405

N 2.339141 0.800882 -0.238575

N 0.531045 -1.259165 -1.001079

N 0.110999 2.605169 0.504676

N -1.828523 0.422492 -0.299177

O 0.104284 1.475504 -2.051877

C 3.297318 1.550246 -0.799350

C 4.632563 1.413506 -0.431002

C 4.966763 0.475319 0.547261

C 3.969019 -0.306094 1.133592

C 2.658423 -0.107231 0.707025

C 1.467843 -0.923197 1.180827

C 1.121025 -1.873168 0.047809

C 1.431153 -3.228943 0.037748

C 1.107639 -3.958627 -1.109696

C 0.496016 -3.322259 -2.189463

C 0.216996 -1.959974 -2.099894

C -0.195533 3.802695 -0.028196

C -0.357413 4.930283 0.759800

C -0.217389 4.805237 2.143258

C 0.094818 3.565872 2.689126

C 0.252360 2.474102 1.838730

C 0.598428 1.104696 2.363661

C -0.975465 -0.706947 1.683751

C -2.130380 -0.221895 0.845493

C -3.453638 -0.443925 1.218750

C -4.472088 0.014561 0.391372

C -4.147838 0.676009 -0.794657

C -2.811254 0.871049 -1.101807

H 2.971528 2.260190 -1.552641

H 5.388850 2.031106 -0.902665

H 5.999784 0.350251 0.856205

H 4.178345 -1.049807 1.895664

H 1.672626 -1.449922 2.110843

H 1.909353 -3.676825 0.902964

H 1.332729 -5.019728 -1.153266

H 0.230857 -3.855750 -3.094826

H -0.260811 -1.416383 -2.907785

H -0.313181 3.819473 -1.105987

H -0.617025 5.877483 0.301347

H -0.379703 5.657540 2.793825

H 0.157771 3.426886 3.761523

H -3.673673 -0.913280 2.169774

H -5.507816 -0.116470 0.684808

H -4.916367 1.062340 -1.454182

H -2.486269 1.396383 -1.992649

H 0.029188 0.927106 3.277005

H 1.660557 1.056652 2.620263

H -1.219742 -0.574508 2.738219

H -0.814503 -1.775224 1.513770

Cl -3.200283 2.517507 3.035093

O -4.591676 2.007326 3.172582

O -2.235161 1.446714 3.506697

O -2.987832 3.748277 3.845392

O -2.909322 2.809623 1.587994

Cl 2.887316 -3.039103 3.664993

O 3.005411 -1.569360 3.981342

O 3.358768 -3.863862 4.795628

O 3.702669 -3.311514 2.422172

O 1.443837 -3.333193 3.344814

N 0.698688 -0.035795 -6.104445

C -0.346842 -0.873419 -5.757557

C -2.517034 -2.606320 -5.041045

C -0.322048 -2.254654 -6.062306

C -1.487832 -0.387492 -5.075817

C -1.383511 -3.085345 -5.710305

C -2.535025 -1.240422 -4.733502

C 1.833720 -0.558696 -6.840736

C 0.664013 1.365220 -5.721990

C -3.679615 -3.509548 -4.703798

H 0.526874 -2.685477 -6.579075

H -1.558851 0.660313 -4.811901

H -1.326281 -4.139685 -5.972855

H -3.394058 -0.824298 -4.211503

H 1.528664 -1.013540 -7.792990

H 2.388575 -1.317851 -6.269919

H 2.521620 0.256491 -7.067633

H -0.173671 1.900340 -6.192022

H 1.587844 1.846442 -6.045635

H 0.577229 1.489356 -4.633743

H -4.194730 -3.179469 -3.796112

H -4.424971 -3.526269 -5.509245

H -3.354083 -4.542632 -4.546364

**^5^TS**

Fe -0.302021 0.796027 -0.619951

N -0.146210 0.158809 1.528322

N 1.885344 0.688748 -0.235689

N -0.031534 -1.408836 -0.708862

N -0.258063 2.675739 0.376801

N -2.346501 0.490439 -0.107638

O -0.433568 1.275711 -2.255534

C 2.865908 1.342760 -0.872583

C 4.198393 1.217050 -0.491880

C 4.509055 0.388001 0.587228

C 3.490159 -0.291055 1.256650

C 2.182108 -0.108733 0.809681

C 0.977896 -0.828273 1.405155

C 0.570665 -1.894229 0.395743

C 0.843515 -3.251536 0.558852

C 0.466381 -4.120292 -0.466003

C -0.161596 -3.616621 -1.605972

C -0.394608 -2.247089 -1.690207

C -0.518202 3.838473 -0.250881

C -0.605629 5.040172 0.432268

C -0.438438 5.031673 1.818459

C -0.175151 3.830473 2.463488

C -0.090770 2.658531 1.711288

C 0.205476 1.333827 2.379825

C -1.430498 -0.474503 1.947573

C -2.604027 -0.007156 1.115795

C -3.910546 -0.109959 1.592216

C -4.960046 0.316982 0.788748

C -4.683258 0.832604 -0.478706

C -3.361405 0.913158 -0.884397

H 2.558062 1.974887 -1.699664

H 4.969293 1.759860 -1.028031

H 5.538313 0.271261 0.911949

H 3.682839 -0.939737 2.104815

H 1.225035 -1.277615 2.367487

H 1.334052 -3.596308 1.463193

H 0.660999 -5.183965 -0.369600

H -0.468372 -4.267415 -2.417558

H -0.877842 -1.798288 -2.551898

H -0.660730 3.766288 -1.323645

H -0.829689 5.954891 -0.104761

H -0.542948 5.945012 2.394013

H -0.092866 3.782850 3.542762

H -4.087791 -0.459532 2.601983

H -5.977545 0.282075 1.162071

H -5.473160 1.199381 -1.124403

H -3.070325 1.335867 -1.839797

H -0.351832 1.301999 3.318043

H 1.269168 1.284587 2.633066

H -1.640371 -0.261294 2.997808

H -1.334807 -1.560385 1.853523

Cl -3.524254 3.006966 3.049938

O -4.932805 2.570353 3.266308

O -2.592235 1.944805 3.596384

O -3.246258 4.297283 3.740116

O -3.263762 3.156149 1.576384

Cl 2.550150 -2.843781 4.008890

O 2.744954 -1.361542 4.200016

O 3.060881 -3.596732 5.174402

O 3.275663 -3.250799 2.748833

O 1.079819 -3.103263 3.804263

N -0.064067 0.438017 -5.570246

C -0.995539 -0.595454 -5.695915

C -2.911596 -2.676807 -5.974987

C -0.789799 -1.651272 -6.607220

C -2.168874 -0.609313 -4.911482

C -1.734989 -2.662717 -6.736661

C -3.098249 -1.632375 -5.057050

C 1.294247 0.246915 -6.068663

C -0.367184 1.611384 -4.841712

C -3.946074 -3.760311 -6.148891

H 0.095854 -1.677134 -7.230662

H -2.326517 0.151609 -4.157129

H -1.553620 -3.459564 -7.452889

H -3.987575 -1.623731 -4.432394

H 1.328246 0.312994 -7.163065

H 1.688012 -0.727437 -5.764647

H 1.935676 1.026608 -5.659056

H -1.382263 1.965922 -5.037193

H 0.361191 2.396101 -5.048027

H -0.340747 1.427798 -3.639345

H -4.480304 -3.958406 -5.215150

H -4.696165 -3.472581 -6.896182

H -3.493603 -4.696567 -6.487773

**^5^IM**

Fe -0.191848 -0.658763 0.782097

N 1.459020 0.519942 -0.204885

N -0.054663 1.227700 1.966392

N -1.234110 0.720014 -0.619121

N 1.518277 -1.333404 1.848786

N 0.264643 -1.881860 -0.888495

O -1.509060 -1.574686 1.584170

C -0.412621 1.422984 3.243493

C -0.231320 2.648322 3.877245

C 0.346674 3.693855 3.154694

C 0.728536 3.492659 1.827856

C 0.510532 2.233431 1.269117

C 0.833585 1.884287 -0.179038

C -0.498360 1.810107 -0.916506

C -0.954201 2.799047 -1.787127

C -2.214204 2.631492 -2.363692

C -2.970395 1.498055 -2.061086

C -2.440650 0.557889 -1.181822

C 1.471992 -2.389237 2.684685

C 2.613919 -2.913095 3.268182

C 3.846276 -2.335430 2.956965

C 3.889914 -1.248848 2.092501

C 2.700323 -0.766103 1.548464

C 2.698292 0.430855 0.622514

C 1.669288 0.002088 -1.589300

C 1.171134 -1.415895 -1.766291

C 1.624031 -2.206953 -2.821324

C 1.132321 -3.498839 -2.957885

C 0.190246 -3.972683 -2.042806

C -0.211660 -3.135836 -1.014860

H -0.847795 0.569266 3.754252

H -0.529385 2.773275 4.912624

H 0.507416 4.660955 3.620819

H 1.190503 4.272520 1.231444

H 1.485202 2.635833 -0.627079

H -0.328475 3.660138 -1.996484

H -2.596511 3.381609 -3.049082

H -3.950885 1.335347 -2.495073

H -2.983243 -0.342397 -0.913034

H 0.484746 -2.802576 2.862057

H 2.541699 -3.772256 3.925292

H 4.766444 -2.745993 3.358178

H 4.834851 -0.814138 1.789869

H 2.395676 -1.830775 -3.481949

H 1.504896 -4.139438 -3.749683

H -0.201324 -4.981572 -2.105498

H -0.921417 -3.445502 -0.255147

H 3.580908 0.369569 -0.017127

H 2.793144 1.344565 1.217503

H 2.729505 0.025609 -1.848769

H 1.148851 0.656957 -2.294815

Cl 4.285935 -3.137300 -0.799647

O 4.411379 -3.885751 -2.081532

O 4.256545 -1.651925 -1.099526

O 5.430887 -3.422967 0.109539

O 2.997086 -3.511247 -0.121117

Cl 2.118976 5.087159 -1.392147

O 2.935133 4.502834 -0.267885

O 2.771222 6.292269 -1.946541

O 0.749928 5.420650 -0.849359

O 1.953715 4.029426 -2.452993

N -4.747206 -0.024315 1.868132

C -5.393726 -0.642776 0.777343

C -6.705954 -1.903086 -1.401751

C -6.444172 0.001692 0.102071

C -4.993306 -1.919160 0.337737

C -7.080037 -0.625615 -0.966738

C -5.646441 -2.528369 -0.728918

C -4.804023 1.430994 2.000207

C -4.030156 -0.748865 2.786511

C -7.423421 -2.588798 -2.538600

H -6.785242 0.981116 0.417067

H -4.139788 -2.406553 0.795042

H -7.894219 -0.108312 -1.467365

H -5.311836 -3.509712 -1.055230

H -5.753822 1.755887 2.441183

H -4.684902 1.905412 1.023504

H -3.990556 1.760005 2.647687

H -4.319412 -1.783539 2.933496

H -3.672786 -0.197111 3.647249

H -2.350323 -1.280699 2.026796

H -6.735567 -3.186967 -3.143934

H -8.199527 -3.268251 -2.165050

H -7.913168 -1.866419 -3.197497

**^5^PC**

Fe -0.431142 -0.230009 0.525021

N 1.604850 0.089463 -0.167472

N 0.319269 1.440593 1.940105

N -0.667030 1.520692 -0.846093

N 0.578374 -1.615044 1.798307

N -0.437689 -1.589528 -1.175786

O -2.538613 -0.487263 1.014648

C 0.022467 1.800042 3.197788

C 0.725434 2.782912 3.888170

C 1.790163 3.412969 3.244028

C 2.124036 3.036694 1.943528

C 1.363614 2.040123 1.328631

C 1.640497 1.588304 -0.104013

C 0.520994 2.147589 -0.974688

C 0.693773 3.254484 -1.806859

C -0.407335 3.708490 -2.532297

C -1.633449 3.052681 -2.410378

C -1.718760 1.959550 -1.554483

C -0.002462 -2.546522 2.578469

C 0.724980 -3.500225 3.269807

C 2.113958 -3.504832 3.127612

C 2.714513 -2.551269 2.317193

C 1.917551 -1.613468 1.658661

C 2.559471 -0.560745 0.774572

C 1.721905 -0.443886 -1.554249

C 0.708990 -1.528335 -1.881438

C 0.974664 -2.424385 -2.918793

C 0.041630 -3.403150 -3.232035

C -1.142781 -3.468293 -2.496950

C -1.328449 -2.553349 -1.474480

H -0.801431 1.271439 3.670127

H 0.445567 3.038410 4.904537

H 2.364650 4.182719 3.749768

H 2.955359 3.483686 1.407732

H 2.604619 1.971640 -0.437226

H 1.667435 3.729317 -1.873553

H -0.303522 4.563964 -3.192444

H -2.506394 3.374471 -2.967833

H -2.649974 1.415334 -1.426115

H -1.087154 -2.516576 2.626829

H 0.215277 -4.240156 3.876365

H 2.717863 -4.262425 3.614803

H 3.783755 -2.562632 2.142768

H 1.929266 -2.383368 -3.429341

H 0.252223 -4.127767 -4.010652

H -1.886624 -4.233862 -2.686126

H -2.214007 -2.601402 -0.845514

H 3.366984 -1.043687 0.220362

H 3.018600 0.207255 1.404786

H 2.721204 -0.851657 -1.722597

H 1.596603 0.383040 -2.259856

Cl 2.506383 -4.499485 -0.569228

O 2.437499 -5.263051 -1.846810

O 3.200062 -3.177496 -0.821478

O 3.262831 -5.254407 0.469351

O 1.113796 -4.219241 -0.073618

Cl 4.467631 3.678252 -1.097220

O 4.759292 2.787053 0.082817

O 5.690331 4.379846 -1.542404

O 3.404644 4.667992 -0.680753

O 3.902110 2.827340 -2.203780

N -4.298836 1.102262 1.559775

C -5.382137 0.786589 0.708074

C -7.588587 0.176258 -1.002218

C -6.132130 1.804293 0.092033

C -5.753544 -0.546675 0.437260

C -7.208059 1.495840 -0.738273
